# Coupling Protein Side-Chain and Backbone Flexibility Improves the Re-design of Protein-Ligand Specificity

**DOI:** 10.1371/journal.pcbi.1004335

**Published:** 2015-09-23

**Authors:** Noah Ollikainen, René M. de Jong, Tanja Kortemme

**Affiliations:** 1 Graduate Program in Bioinformatics, University of California San Francisco, San Francisco, California, United States of America; 2 DSM Biotechnology Center, Alexander Fleminglaan 1, Delft, The Netherlands; 3 California Institute for Quantitative Biosciences (QB3), University of California San Francisco, San Francisco, California, United States of America; 4 Department of Bioengineering and Therapeutic Science, University of California San Francisco, San Francisco, California, United States of America; Iowa State University, UNITED STATES

## Abstract

Interactions between small molecules and proteins play critical roles in regulating and facilitating diverse biological functions, yet our ability to accurately re-engineer the specificity of these interactions using computational approaches has been limited. One main difficulty, in addition to inaccuracies in energy functions, is the exquisite sensitivity of protein–ligand interactions to subtle conformational changes, coupled with the computational problem of sampling the large conformational search space of degrees of freedom of ligands, amino acid side chains, and the protein backbone. Here, we describe two benchmarks for evaluating the accuracy of computational approaches for re-engineering protein-ligand interactions: (i) prediction of enzyme specificity altering mutations and (ii) prediction of sequence tolerance in ligand binding sites. After finding that current state-of-the-art “fixed backbone” design methods perform poorly on these tests, we develop a new “coupled moves” design method in the program Rosetta that couples changes to protein sequence with alterations in both protein side-chain and protein backbone conformations, and allows for changes in ligand rigid-body and torsion degrees of freedom. We show significantly increased accuracy in both predicting ligand specificity altering mutations and binding site sequences. These methodological improvements should be useful for many applications of protein – ligand design. The approach also provides insights into the role of subtle conformational adjustments that enable functional changes not only in engineering applications but also in natural protein evolution.

## Author Summary

Designing new protein–ligand interactions has tremendous potential for engineering sensitive biosensors for diagnostics or new enzymes useful in biotechnology, but these applications are extremely challenging, both because of inaccuracies of the energy functions used in modeling and design, and because protein active and binding sites are highly sensitive to subtle changes in structure. Here we describe a new method that addresses the second problem and couples changes in the structure of the protein backbone and of the amino acid side chains, the amino acid sequence, and the conformation of the ligand and its orientation in the binding site. We show that our method improvements significantly increase the accuracy of designing protein–ligand interactions compared to current state-of-the-art design methods. We assess these improvements in two important tests: the first predicts mutations that change ligand-binding preferences in enzymes, and the second predicts protein sequences that bind a given ligand. In these tests, subtle conformational changes made in our model are essential to recapitulate both the results from engineering experiments and the sequence diversity occurring in natural protein families. These results therefore shed light on the mechanisms of how new protein functions might have emerged and can be engineered in the laboratory.

## Introduction

Interactions between small molecules and proteins play critical roles in essentially all biological processes. Naturally occurring proteins have evolved to function as sensitive small-molecule sensors that detect and respond to changes in the extra- and intracellular environment, or as catalysts that enhance the speed of chemical reactions by orders of magnitude. To harness these capabilities, both industry and medicine take advantage not only of existing proteins, but increasingly utilize strategies to reengineer proteins to function with altered ligands, cofactors and substrates. These approaches have tremendous potential for expanding the range of accessible biological functions to produce industrially or therapeutically valuable compounds, develop new biosensors as research tools or for medical diagnostics, or detect and respond to harmful compounds. Metabolic pathway engineering requires fine-tuning enzyme activity and specificity to optimize the production of small molecule products such as drugs or biofuels [1]. Enzyme specificity is also important in therapeutic strategies such as suicide gene therapy, in which a therapeutic enzyme must convert a specific pro-drug into a cytotoxic compound in order to selectively kill cancer cells [2,3], in food manufacturing to achieve the desired taste and appearance of food products [4], and in bioremediation to specifically degrade target toxic pollutants [5].

Despite the growing number of potential applications for reengineering protein-ligand specificity, our ability to accurately predict the required amino acid sequence changes has been limited [6]. Most approaches to enzyme engineering have used screening strategies based on structural and chemical intuition, or employed the power of directed evolution [7,8]. Accurate computational design methods would not only complement these strategies but could also enable applications that are otherwise limited by experimental throughput or lack of a starting activity for a desired new substrate. Moreover, the ability to predict specificity changes would be a stringent test of the accuracy of computational methods, and, if successful, would provide insights into the mechanistic basis and the evolution of protein specificity.

Previous work on applying computational methods to design specificity has focused largely on interactions between proteins, although there are examples of applications to enzymes [9–11]. Computational methods to re-engineer protein–protein specificity have typically employed a “second-site suppressor” strategy, in which a mutation is made on one protein to destabilize its interaction with a binding partner, and a second compensating mutation is made on the binding partner to re-stabilize the interaction [12]. This approach has been successfully applied to re-design the specificity of a number of proteins, including interactions between PDZ domains and their binding peptides [13], a DNase–inhibitor pair [12,14], a small GTPase and its guanine exchange factor [15], and the interaction between a ubiquitin ligase and a ubiquitin-conjugating enzyme [16]. In the majority of these studies, protein–protein interactions are modeled as rigid complexes and are not allowed to re-orient relative to each other during the sequence design, although these approaches have been explored in a few cases [14,17].

Modeling interactions between proteins and small molecules requires in addition sampling of ligand degrees of freedom, including ligand rotation and translation as well as the conformational flexibility of the small molecule. These degrees of freedom need to be sampled accurately because enzymes are highly sensitive to subtle changes in the conformations of their active sites [18], making the design of enzyme specificity a particularly challenging problem. Previous work has demonstrated the importance of high-resolution sampling of both amino acid side-chain and small molecule conformational flexibility to achieve accurate placement of small molecules in enzyme active sites [19]. Similar high-resolution sampling has enabled computational protein design methods to recapitulate the native sequences of ligand binding and enzyme active sites [20–22] and to predict the effect of mutations on ligand binding [23].

A common feature of the previous work in this area is the assumption that the protein backbone remains fixed in conformation during the sequence design step, although there are some exceptions [9,24]. The fixed backbone approximation is mainly made for computational efficiency. However, changes in the protein backbones to accommodate changes in amino acid sequence [25] are the rule rather than the exception, and a key reason for failed designs is that they do not adopt the required precise geometry of an engineered functional site [6,18]. In support of these ideas, sampling protein backbone flexibility has been shown to improve the accuracy of computational approaches to model and design proteins as well as protein–protein interactions [25–29].

Given these observations, we reasoned that incorporating backbone flexibility might also improve the accuracy of designing interactions between proteins and small molecules. To test this idea, we first created a computational benchmark to evaluate the ability of protein design methods to re-design enzyme substrate specificity. We then used this benchmark to show that a new method that couples backbone flexibility with changes in amino acid side-chain conformations, allowing subtle rearrangements of the active site, resulted in a 5.75-fold increase in the percent of correct predictions over a state-of-the-art protein design method that assumes a fixed backbone. The fixed backbone method and the new approach, which we refer to as “coupled moves”, are both implemented in the protein modeling and design software Rosetta [30] and use an identical energy function, thereby evaluating the influence of improved conformational sampling. Next, we created a second benchmark that tests how well a given design method can recapitulate the set of naturally occurring ligand binding site sequences in eight families of co-factor binding domains. We found a significant increase in the recapitulation of natural ligand binding site sequences using the coupled moves method relative to fixed backbone design, suggesting that the coupled moves method increases the accuracy of the design of sequence libraries for protein–ligand binding sites. Taken together, these results highlight the importance of allowing subtle conformational changes in protein backbones and provide new algorithms and benchmarks for improving the accuracy of modeling and designing protein–ligand interactions. Moreover, our results provide insights into how subtle coupled side-chain and backbone conformational changes enable sequence changes that either change or maintain an existing function.

## Results

### Evaluating the accuracy of computational re-design of enzyme specificity

To evaluate how accurately a given computational protein design method could predict mutations that change enzyme specificity, we required a set of known specificity altering mutations that have been experimentally characterized both structurally and biochemically. We sought mutations and enzymes that satisfied the following criteria: 1) there exists a co-crystal structure of the wild-type enzyme bound to the native substrate (using an inactive enzyme version or a substrate-analog) and a co-crystal structure of the mutant enzyme bound to the non-native substrate/substrate analog, 2) the native and non-native ligands share a common substructure that can be used for superimposition, 3) the mutations are located in the active site within 6Å of the ligand and do not occur at positions that are critical for the chemical step of the reaction the enzyme catalyzes.

**Fig 1:**
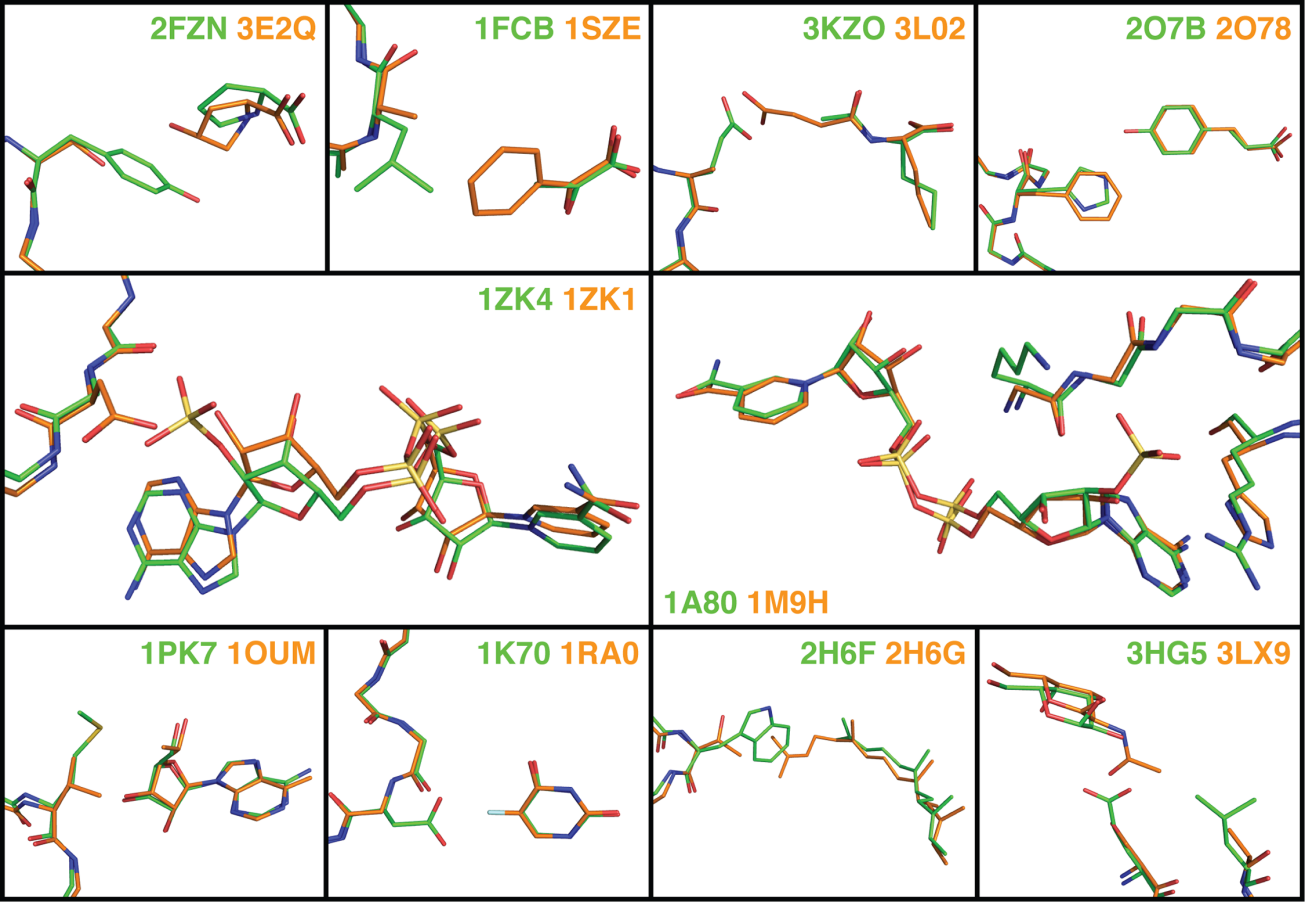
Structures of wild-type and mutant binding sites for known specificity altering mutations. Close-up images of the substrate binding sites for the ten enzymes in our benchmark with known specificity altering mutations are shown in stick representation. The PDB IDs of the wild-type (green) and mutant (orange) structures are displayed in each panel.

To identify examples that satisfied the above criteria, we used the PDBe database [31] to find all cases of enzymes with solved crystal structures in which the enzyme was bound to its native substrate/substrate analog. We then filtered this set of enzymes to only include examples for which there was at least one structure of the same enzyme bound to a non-native substrate/substrate analog with one or two active site mutations. Finally, we examined the papers associated with the mutant enzyme structures to identify the cases where the specificity of the wild-type and mutant enzymes were experimentally characterized and it had been shown that the mutation(s) alter the specificity of the enzyme to prefer the non-native substrate. This resulted in 10 enzymes with a total of 17 specificity altering mutations (**Table 1**). Structures of the mutant and wild-type substrate binding sites are shown in **Fig 1**. Experimental data on the effect of the mutations on enzyme specificity are shown in **S1 Table.**

**Table 1:** Comparison of fixed backbone and coupled moves methods on predicting specificity altering mutations.

Mutant #	Wild-type PDB ID	Mutant PDB ID	Mutation	# of Designed Positions	Fixed Backbone Percentile	Fixed Backbone Rank	Coupled Moves Percentile	Coupled Moves Rank
1	2FZN	3E2Q	Y540S	2	–	–	95.8	2
2	1FCB	1SZE	L230A	5	–	–	63.0	28
3	3KZO	3L02	E92A	5	78.9	5	100	1
4	3KZO	3L04	E92S	5	–	–	86.5	8
5	3KZO	3L05	E92P	5	–	–	–	–
6	3KZO	3L06	E92V	5	–	–	63.5	20
7	2O7B	2O78	H89F	4	–	–	90.9	3
8	1ZK4	1ZK1	G37D	7	93.8	2	90.2	6
9	1A80	1M9H	K232G	5	–	–	71.4	19
10	1A80	1M9H	R238H	5	–	–	–	–
11	1PK7	1OUM	M64V	3	–	–	69.2	13
12	1K70	1RA0	D314S	4	–	–	63.6	9
13	1K70	1RA5	D314G	4	–	–	90.9	3
14	1K70	1RAK	D314A	4	–	–	72.7	7
15	2H6F	2H6G	W602T	9	–	–	61.3	37
16	3HG5	3LX9	E203S	7	–	–	93.5	7
17	3HG5	3LX9	L206A	7	–	–	87.1	13

Dashes denote cases where the known mutation was not enriched in the predicted sequences using non-native substrate/substrate analogs and therefore not predicted to be a specificity altering mutation. “# of Designed Positions” refers to the number positions that were allowed to mutate in the simulation. “Percentile” refers to the percentile of the known mutation relative to all other predicted mutations when sorted in descending order of their percent enrichment. “Rank” refers to the index of the known mutation in this sorted list. The number of correctly predicted mutations is significantly greater with the coupled moves method than with fixed backbone design (p < 0.0001).

To quantify the extent to which a given design method could recapitulate the known specificity altering mutations, we first predicted the set of “tolerated sequences” for the native ligand and for the non-native ligand. To predict tolerated sequences, we ran design simulations in which a Monte Carlo simulated annealing protocol in Rosetta was used to optimize amino acid sequences and side-chain conformations in a region around the active site, as described in the Methods. For each predicted mutation, we determined whether or not the mutation had a higher percent occurrence in the non-native ligand sequences than in the native ligand sequences. If a known specificity altering mutation had a higher percent occurrence in the non-native ligand sequences, we considered this to be a “correct prediction.” For each mutation, we also computed a “percent enrichment”, which is simply the percent occurrence in the non-native ligand sequences subtracted by the percent occurrence in the native ligand sequences. For each correctly predicted known mutation, we determined how this mutation ranked relative to all other mutations at the positions that were allowed to mutate by sorting all mutations in descending order of their percent enrichment. Finally, we repeated this benchmark in the opposite direction by predicting mutations that would revert the mutant enzyme back to the wild-type enzyme. In these “Mutant to WT” cases, we considered the specificity altering mutation to be a correct prediction if it was enriched in the sequences designed for the native ligand relative to the sequences designed for the non-native ligand.

**Fig 2:**
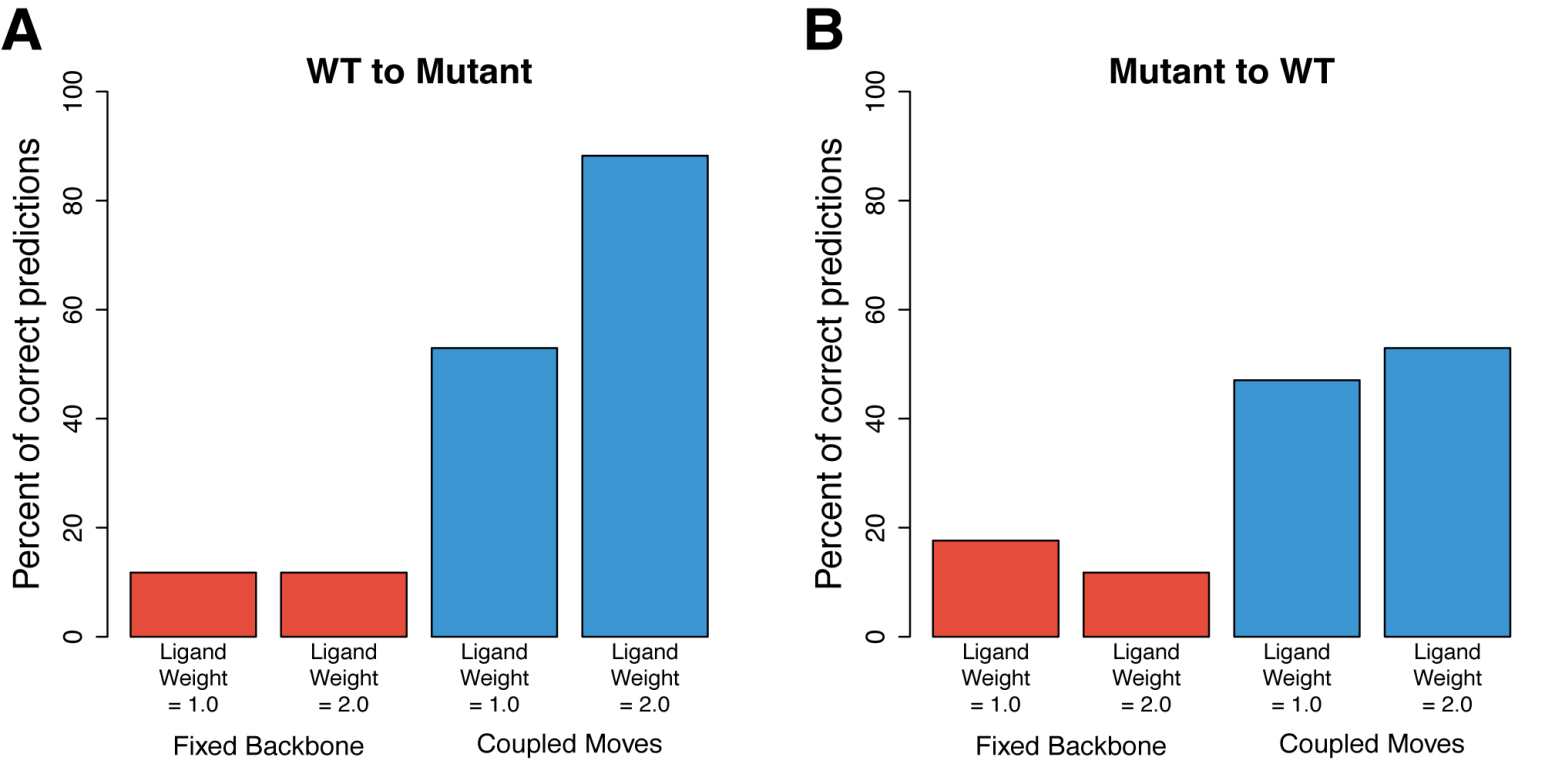
Performance of computational protein design methods on predicting specificity altering mutations. Percent of mutations predicted correctly for specificity altering mutations starting from A) the wild-type structure and B) the mutant structure. Results using fixed backbone design (red) and the coupled moves protocol (blue) are shown where protein–ligand interactions are up-weighted (ligand weight = 2.0) or not up-weighted (ligand weight = 1.0).

We first used this benchmark to test the standard fixed backbone protein design method in the modeling and design program Rosetta [30] on its ability to predict the 17 known specificity altering mutations (Methods). We found that this “fixed backbone” approach could only predict 2 out of the 17 known specificity altering mutations correctly (Table 1, **Fig 2A**). Previous work in modeling peptide-binding specificity found that up-weighting intermolecular interactions relative to intra-molecular interactions improved performance [28]. We therefore repeated the benchmark using a modified score function that up-weighted protein–ligand interactions by a factor of two. While this resulted in different mutations in the benchmark being predicted correctly, it did not change the overall percent of correct predictions (**Fig 2A**). To determine if additional optimization of side-chain conformations could improve the performance of fixed backbone design, we used an algorithm called “min packing”, where side-chain torsions are minimized for each rotamer during every move in the simulation. However, this did not significantly change the percent of correct predictions (**S1 Fig**).

### A coupled moves method to model and design protein–ligand interactions

**Fig 3:**
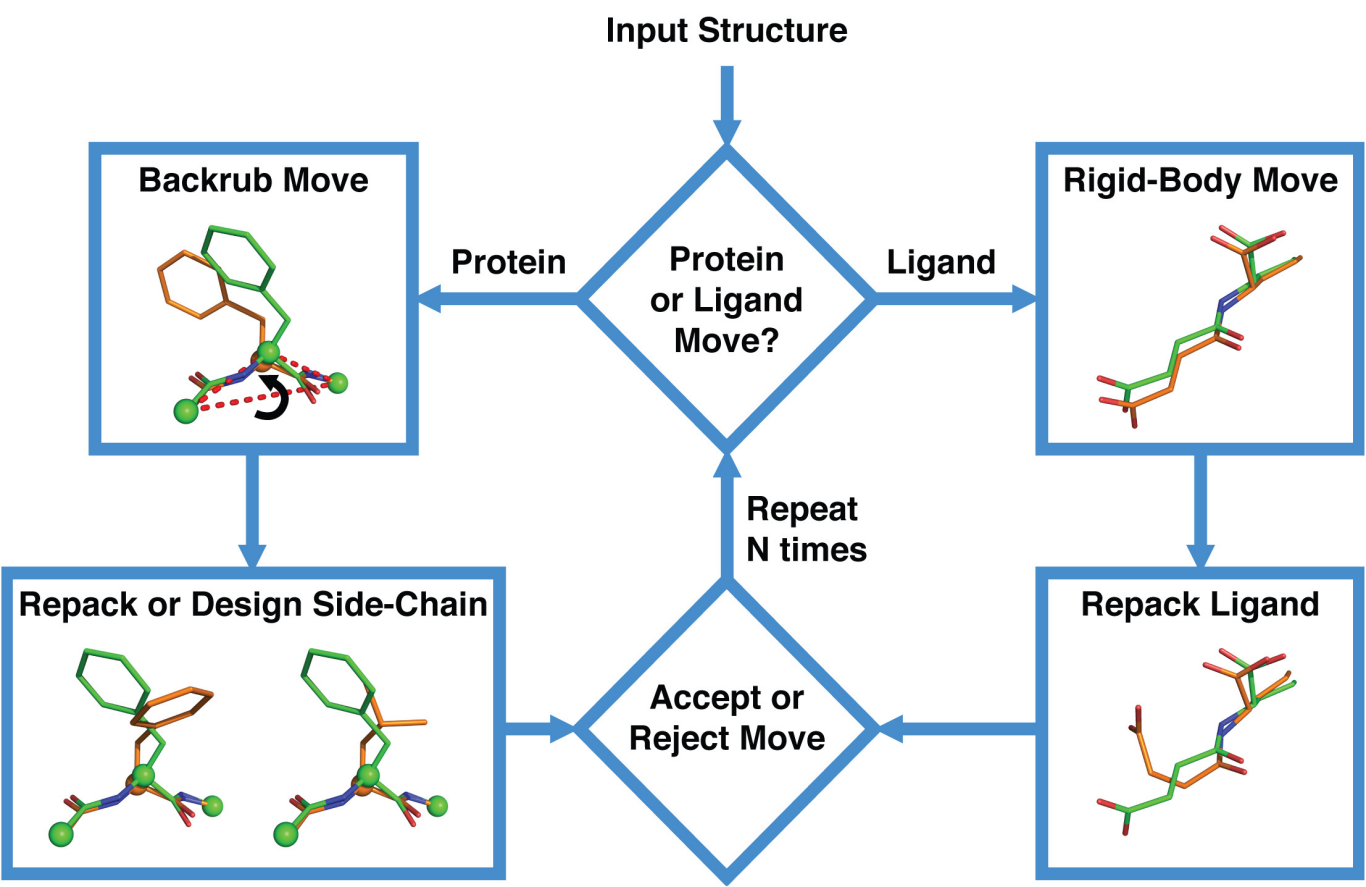
Flowchart outlining the coupled moves method. **The protocol starts with an input structure of a protein–ligand interaction, and performs either coupled protein or ligand moves.** Each protein move involves a backrub move coupled to side-chain repacking or design and each ligand move involves a rigid-body rotation and translation coupled to ligand repacking. A move is either accepted or rejected depending on the change in energy, and a total of N moves are performed, where N can be set by the user.

Fixed backbone and “min packing” simulations showed a surprisingly poor performance on the enzyme specificity design set. To investigate whether a method that allows protein backbone flexibility could improve the accuracy of these predictions, we developed a protein design method that combines backbone, side-chain and ligand flexibility. Our previous approaches to representing protein backbone flexibility first generated an ensemble of backbone conformations and then used fixed backbone design on each member of the ensemble [29]. While this approach improved prediction accuracy in a variety of applications including molecular recognition specificity [29] and amino acid covariation [27], it might not accurately capture how protein backbones respond to sequence mutations as the original backbone ensembles are created with the wild-type sequence. Here, we instead coupled “backrub” moves [32], which locally alter the protein backbone, with changes in amino acid side-chain conformation (repack) and/or amino acid identity (design). We used a similar strategy to model ligand flexibility, where we coupled ligand rotations and translations, which alter the orientation of the ligand relative to the protein, with changes in the ligand internal degrees of freedom. To combine these protein and ligand coupled moves into a single protocol, which we refer to as the “coupled moves” method, we used a Monte Carlo sampling approach illustrated in **Fig 3**.

The coupled moves method is different from previous design methods using “backrub” moves because it enables amino acid mutations and changes in side-chain conformations to occur simultaneously with changes in the protein backbone conformation (previous methods applied backbone and side-chain moves separately [25]). To do this, the new protocol uses a different strategy to decide how to select a mutation or change in side-chain conformation in the context of a given change in backbone conformation. Following a change in backbone conformation, the change in energy of each potential mutation or side-chain conformation on the moved backbone segment is calculated and these energies are used to compute the probability of each potential mutation or side-chain conformation based on a Boltzmann distribution. These probabilities are used to select a mutation or side-chain conformation to couple with the new backbone conformation and the Metropolis criterion [33] is applied to decide whether to accept or reject the coupled move. While “backrub” moves were used to generate new backbone conformations in this study, the coupled moves method is generalizable and other types of backbone movements could be used as well. For example, coupled moves that involve the ligand use a rigid-body rotation and translation in place of a “backrub” move.

The input to the coupled moves method is a structure of a protein–ligand complex and a file that specifies which amino acid positions are allowed to mutate and which positions are allowed to change conformation. For each accepted coupled move that involves a change in amino acid identity, the resulting amino acid sequence of the design residues is saved in a list that is outputted upon completion of the simulation. These sequences can then be further analyzed to choose appropriate mutations for the given design application. Optionally, the lowest energy structure of each unique mutant sequence encountered during the simulation can be saved for structural analysis.

### The coupled moves method improves prediction of enzyme specificity altering mutations compared to fixed backbone design

We implemented the coupled moves method in the Rosetta software suite [30] to enable direct comparison with fixed backbone design using exactly the same energy function. We found that the coupled moves method increased the percent of correct predictions for the known specificity altering mutations 4.5-fold from 12% to 53% compared to fixed backbone design (**Fig 2A**). We also observed a 3.5-fold increase (from 12% to 41%) in the percent of correct predictions when starting from the mutant and trying to predict the wild-type sequence (**Fig 2B**). When we up-weighted the protein–ligand interactions in coupled moves simulations by a factor of two, we observed a further improvement in the percent of correct predictions, from 53% to 88% for specificity altering mutations and from 41% to 47% for wild-type reversion mutations. When combined, the results of these two sets of mutations show that the coupled moves method increased prediction accuracy by 5.75-fold, from 12% to 68%, over fixed backbone design (p < 10^−6^). Up-weighting protein–ligand interactions further did not improve the results (**S2 Fig**).

**Fig 4:**
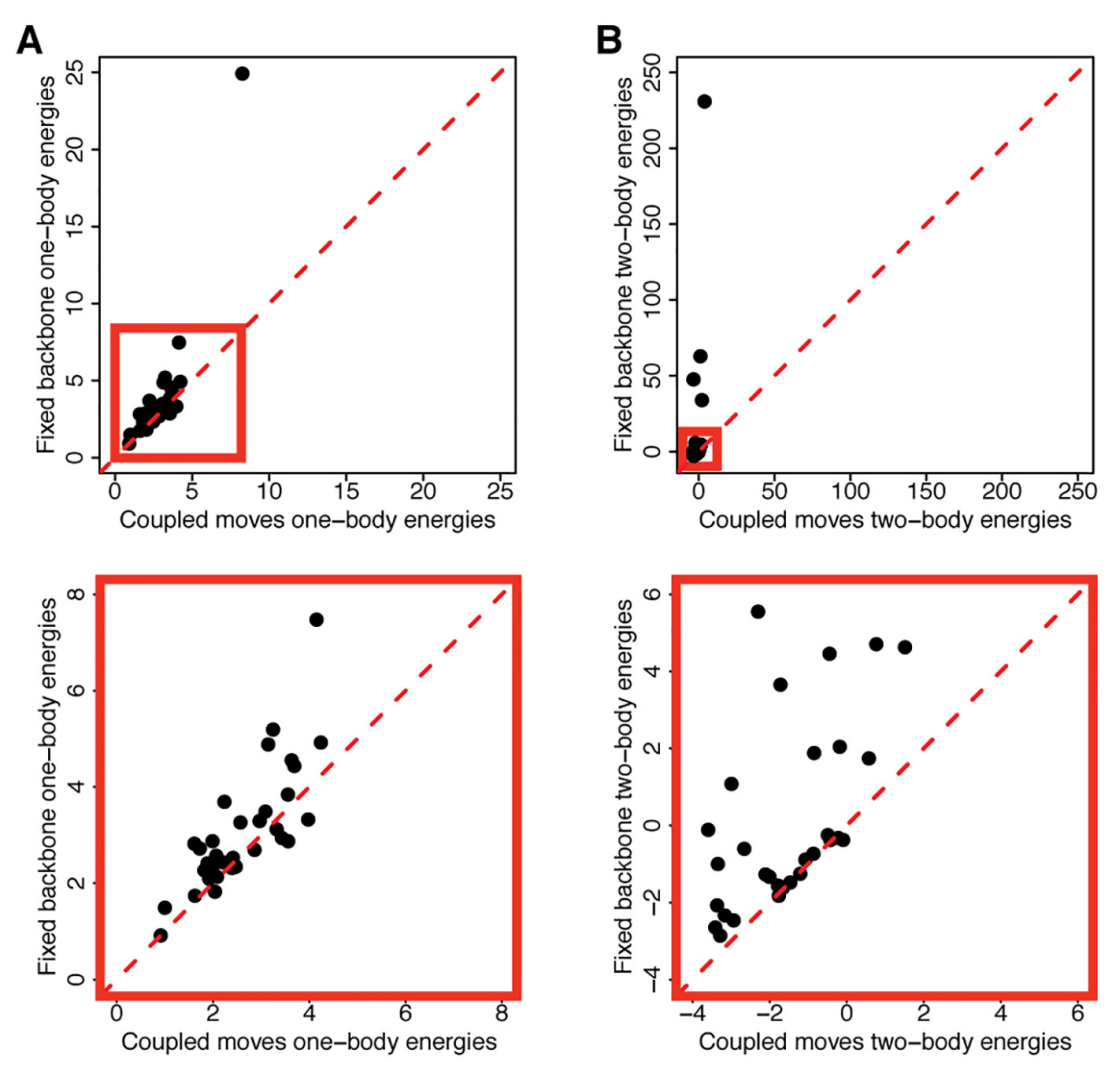
Comparison of the predicted energetic effects of the specificity altering mutations between the fixed backbone and coupled moves methods. Predicted energies (in Rosetta energy units) for each of the specificity altering mutations for A) one-body interactions and B) two-body interactions of the residue at the mutated position. Scatterplots show a comparison of energies from fixed backbone and coupled moves methods, where each dot denotes a mutation and y = x is shown as a dashed red line. Data points above the diagonal indicate larger (more unfavorable) predicted energies using fixed backbone design. The bottom scatterplots show close-ups of the plot area within the red boxes in the top scatterplots.

To understand the basis underlying the improvement in the coupled moves method at predicting specificity altering mutations, we examined structural models of each of the known mutations using the coupled moves method and using fixed backbone design. We first compared these models based on the RMSDs of the mutated residues to the known crystal structures as well as the RMSDs of the neighboring residues, but we did not find a significant difference between the fixed backbone and coupled moves methods for either set of residues (**S3 Fig**). The lack of difference in RMSDs for these methods could be due to the fact that these values are in the range of deviations observed within the ensembles underlying typical X-ray crystal structures [34]. Next, we compared the energetic contribution of the mutations using the coupled moves method and fixed backbone design. We found that using the coupled moves method, the mutations generally obtained lower one-body (intra-residue) and two-body (inter-residue) interaction energies compared to fixed backbone design (**Fig 4**).

**Fig 5:**
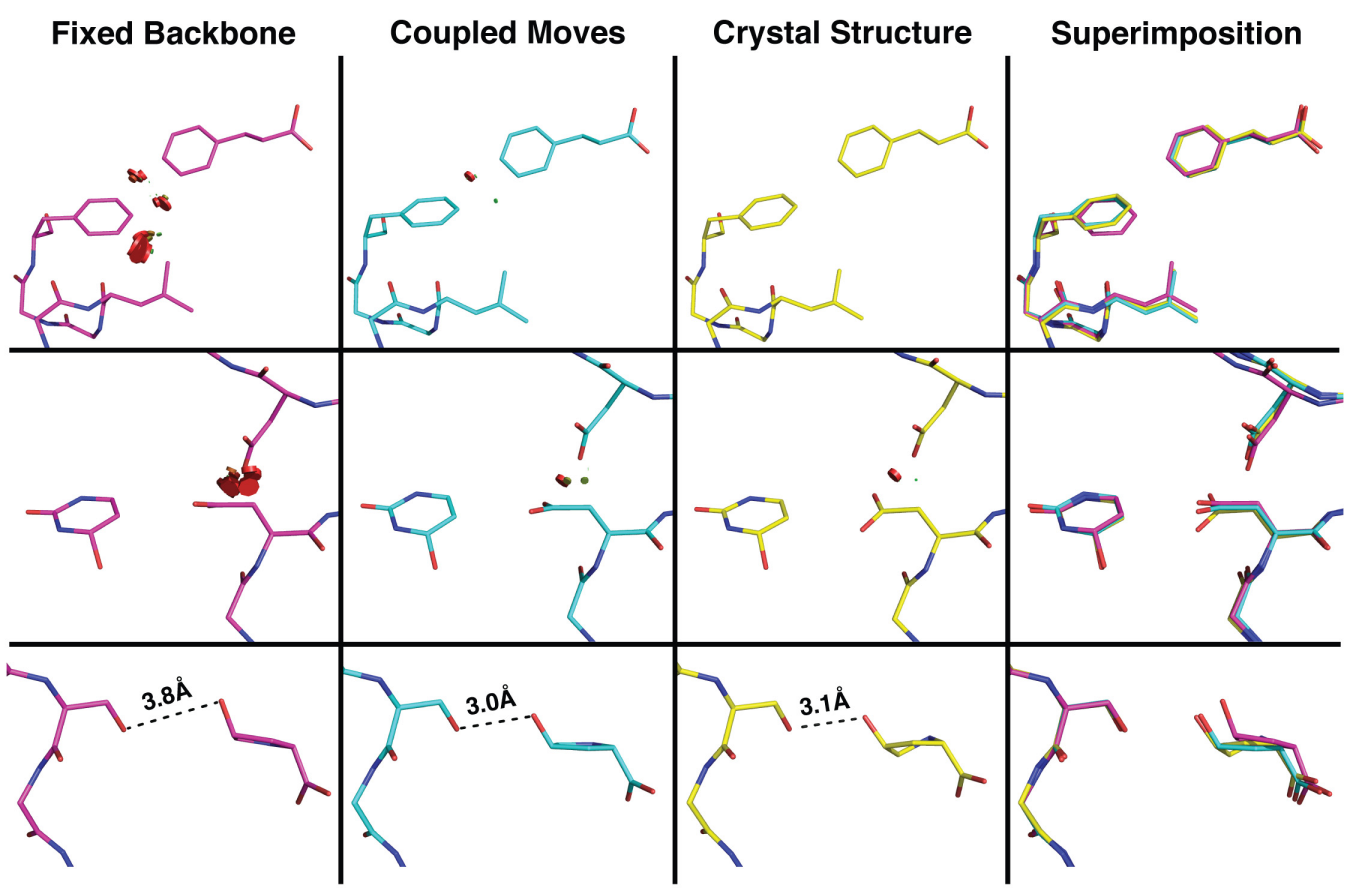
Comparison of models for specificity altering mutations from fixed backbone and coupled moves methods, and crystal structures. Each row displays an example specificity altering mutation from fixed backbone (magenta) or coupled moves (cyan) models, as well as the crystal structure (yellow) and the superimposition of all three (far right column). Red disks denote steric clashes and dashed black lines denote hydrogen-bonding interactions.

Comparing specificity altering mutations modeled using - fixed backbone design or using the coupled moves method revealed that the mutations often produce steric clashes in fixed backbone design models while adopting favorable conformations in the coupled moves method models (**Fig 5**). One reason that this occurs is because backbone flexibility allows neighboring positions to move slightly and make room for the specificity altering mutation, as in the top two rows of **Fig 5**. Sampling ligand rigid-body rotation and translation can also result in more favorable conformations, as in the bottom row of **Fig 5**, where ligand movements are necessary in order to achieve an optimal hydrogen bonding geometry. The findings that structural changes are subtle and often distributed across the environment of the mutated residue are consistent with our observation above that there are no significant differences in the RMSDs to the crystal structure of the mutant when only considering the mutated residues (**S3 Fig**). Overall our results suggest that fixed backbone design is unable to correctly predict many of the specificity altering mutations because it cannot sample low-energy conformations that require backbone movements. In fact, min packing, which minimizes side-chain conformations on a fixed backbone, still fails to identify many of the correct mutations (**S1 Fig**). In contrast, the coupled moves method makes subtle changes in backbone and ligand conformations that allow better optimization of steric packing and other interactions that are sensitive to precise geometries, such as hydrogen bonding.

### Predicting sequence tolerance in ligand binding sites

In most cases, the known specificity altering mutation was not the highest-ranking mutation predicted to change enzyme specificity (**Table 1**). This is likely due to inaccuracies in the design method, such as errors in the energy function used for ranking. However, an alternative explanation is that some of the higher-ranked mutations could be functional but were simply not tested experimentally. This observation raises the following question: how accurate is the overall set of ligand binding site sequences predicted by the coupled moves method, or more generally, by any given protein design approach? To address this question, we needed a set of known ligand binding site sequences to use as a gold standard by which to compare sequences predicted by a given design method. To obtain these sequences, we sought protein families that satisfied the following criteria: 1) the protein family has at least one representative crystal structure bound to the cognate ligand to use as input for design, 2) the protein family has a large number of diverse sequences such that the binding site is not completely conserved, 3) all members of the protein family are capable of binding the cognate ligand using the same ligand binding site.

We took advantage of two existing resources to find protein families that satisfied the above criteria: the Protein Data Bank (PDB) [35], which provides thousands of examples of specific proteins bound to small molecule ligands, and Pfam [36], which groups all known proteins into families based on their sequences and assigns each family a unique ID. We used these resources to create a mapping between protein families and the small molecule ligands that the family members are known to bind. Using this protein family to ligand mapping, we found that the protein families with the greatest number of unique proteins bound to the same ligand tended to be protein domains of enzymes that are responsible for binding small molecule co-factors. We reasoned from these results that co-factor binding domains would be ideal systems for our benchmark, given that enzymes containing these domains require binding to a specific small molecule co-factor in order to function and this requirement is likely to be conserved throughout the domain family. This benchmark is thus conceptually different from previous sets [22] that included complexes between proteins and small-molecule inhibitors. In these cases it is not guaranteed that other protein family members would bind the same inhibitor and could thus be used to evaluate not just a single “native” but also the set of “tolerated” sequences.

We selected a set of co-factor binding protein families that had the greatest number of available sequences and non-redundant co-factors, resulting in the 8 families shown in **Table 2**. For each protein family, we used the highest resolution crystal structure bound to the cognate co-factor to identify ligand binding site positions used as input for design. Ligand binding sites were defined as any amino acid position with a side-chain heavy atom within 6Å of any heavy atom of the co-factor ligand. Natural sequences of these binding sites were obtained using the protein family alignment from Pfam and filtered to remove all redundant sequences. Ligand binding site positions were allowed to mutate to any amino acid during design and neighboring positions were allowed to repack. The resulting predicted design sequences were compared to the natural sequences by calculating the Jensen–Shannon divergence at each position and subtracting this value from one, which we refer to as “profile similarity” (see Methods). This value represents the similarity in the amino acid distributions between the natural and predicted sequences at a given position.

**Table 2:** Comparison of fixed backbone and coupled moves methods on predicting co-factor binding site sequences.

Protein Domain	PFAM ID	Co-factor ligand	# of unique binding site sequences	PDB ID	Number of designed positions	Fixed Backbone Mean Profile Similarity	Coupled Moves Mean Profile Similarity
Cytochrome P450	PF00067	Heme	8296	2IJ2	30	0.233	0.312
Methyltransferase domain	PF08241	S-adenosyl methionine	7042	3DLC	19	0.443	0.568
Acetyltransferase (GNAT) family	PF00583	Coenzyme A	4084	3S6F	14	0.541	0.639
Glutathione S-transferase	PF13417	Glutathione	2948	3R2Q	11	0.540	0.637
Short chain dehydrogenase	PF00106	NAD	21085	1ZK4	21	0.541	0.659
Aminotransferase class I and II	PF00155	Pyridoxal 5'-phosphate	3149	2XBN	14	0.401	0.544
FAD dependent oxidoreductase	PF01266	FAD	3053	3DK9	30	0.608	0.683
Flavodoxin	PF00258	FMN	947	1F4P	19	0.438	0.637

Sequence alignments of naturally occurring co-factor binding domains were taken from Pfam and filtered for redundancy. Positions were included in design if they had a side-chain heavy-atom within 6Å of the co-factor ligand and no gaps in the multiple sequence alignment.

### The coupled moves method improves prediction of sequence tolerance in ligand binding sites compared to fixed backbone design

**Fig 6:**
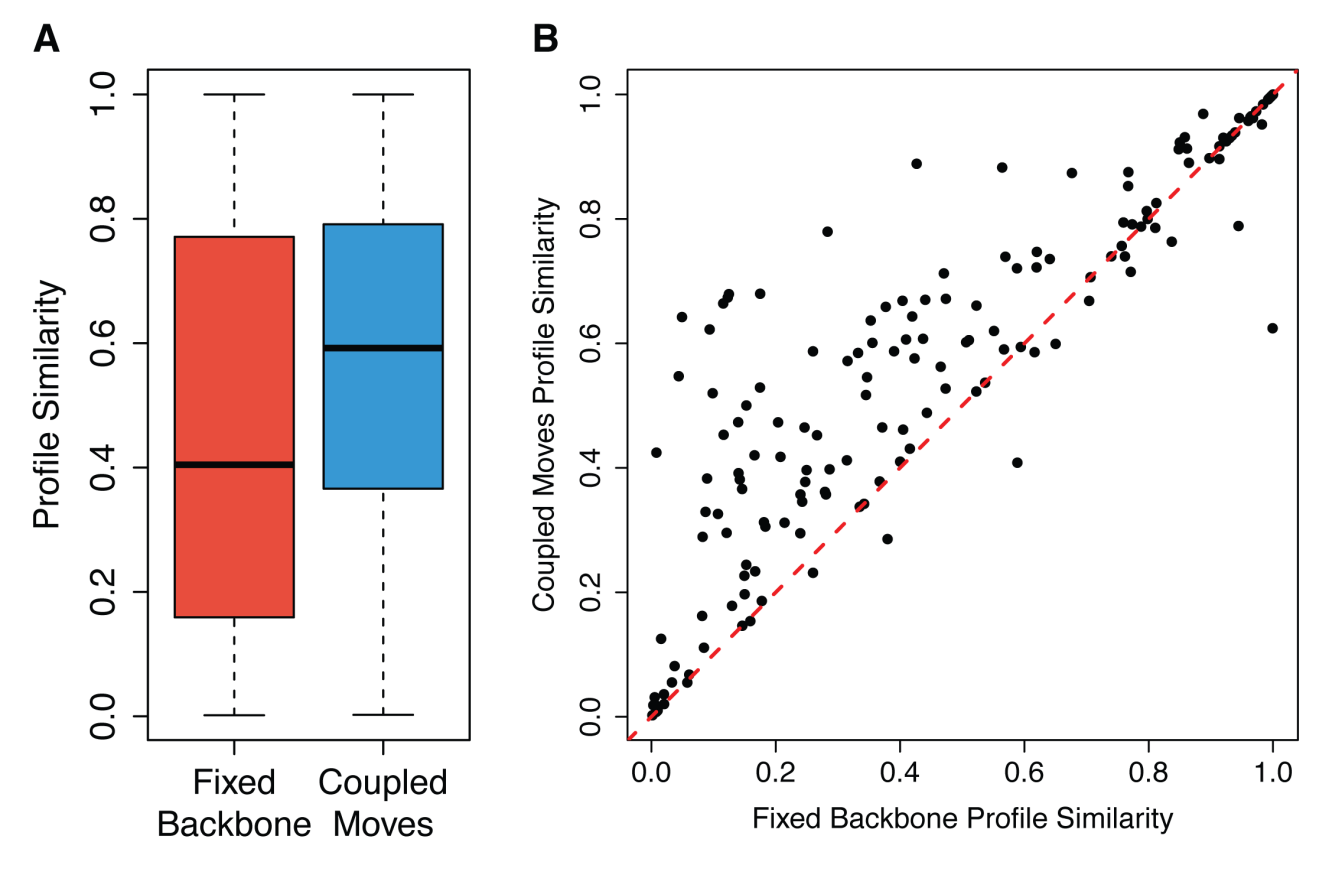
Performance of computational protein design methods on predicting ligand binding site sequences. A) Boxplot of distributions of profile similarity values between natural and designed sequences for each of the 158 positions in 8 co-factor binding sites. Whiskers denote minimum and maximum, top and bottom of the box indicate 75th and 25th percentile, respectively, and the bold line shows the median. B) Scatterplot comparing profile similarity for each position in sequences designed with fixed backbone and coupled moves methods. y = x is shown as a dashed red line. Data points above the diagonal indicate improved predictions using the coupled moves method.

**Fig 7:**
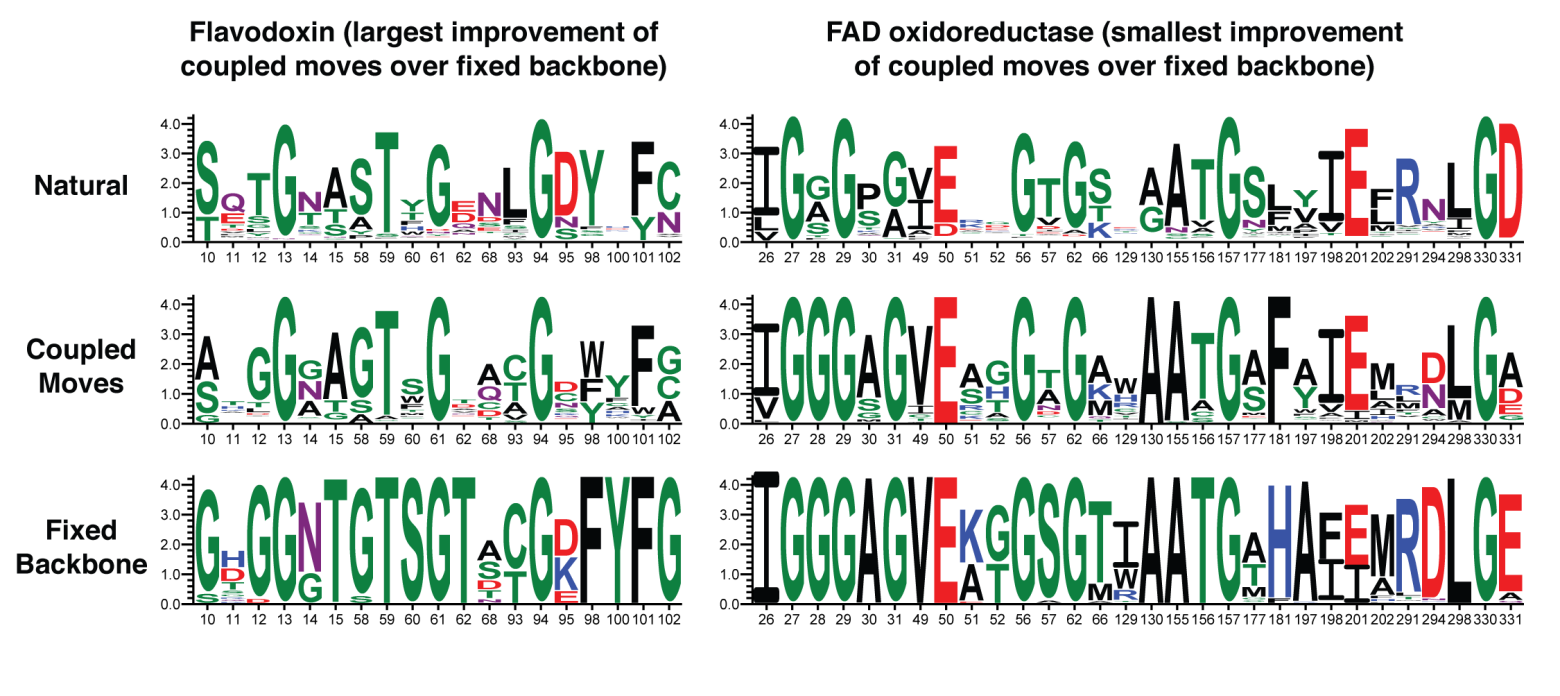
Sequence logos for predicted and naturally occurring binding site sequences. Two representative examples showing the largest (left) and the smallest (right) improvement of coupled moves (middle row) over fixed backbone design (bottom row) with respect to profile similarity with natural sequences (top row). The height of the letter representing each amino acid corresponds to its frequency and the height of each column is inversely proportional to the sequence variation at that position.

We applied the coupled moves method to predict the set of tolerated sequences in ligand binding sites for each of the 8 co-factor binding protein families and calculated profile similarity with the natural sequences at each position. For comparison, we also used fixed backbone design to generate the same number of total sequences as obtained from the coupled moves simulations. The resulting profile similarity distributions for coupled moves and fixed backbone design across the 158 ligand binding site positions in 8 protein families are shown as boxplots in **Fig 6A**. The coupled moves method increased the median profile similarity relative to fixed backbone design from 0.40 to 0.59 (p < 10^−11^). To understand how the fixed backbone and coupled moves methods affect the profile similarity score for each position individually, we compared the values for the 158 positions for each method, as shown in **Fig 6B**. Data points above the diagonal indicate the cases where the coupled moves method performs better. Sequence logos for predicted and naturally occurring co-factor binding site sequences are shown in **Fig 7** for the domains where the coupled moves method had the greatest and smallest improvements over fixed backbone design. The remaining sequence logos are shown in **S4**–S**9** **Figs**. From the results on this benchmark it is clear that the coupled moves method improved the prediction of sequence tolerance in ligand binding sites relative to fixed backbone design.

**Fig 8:**
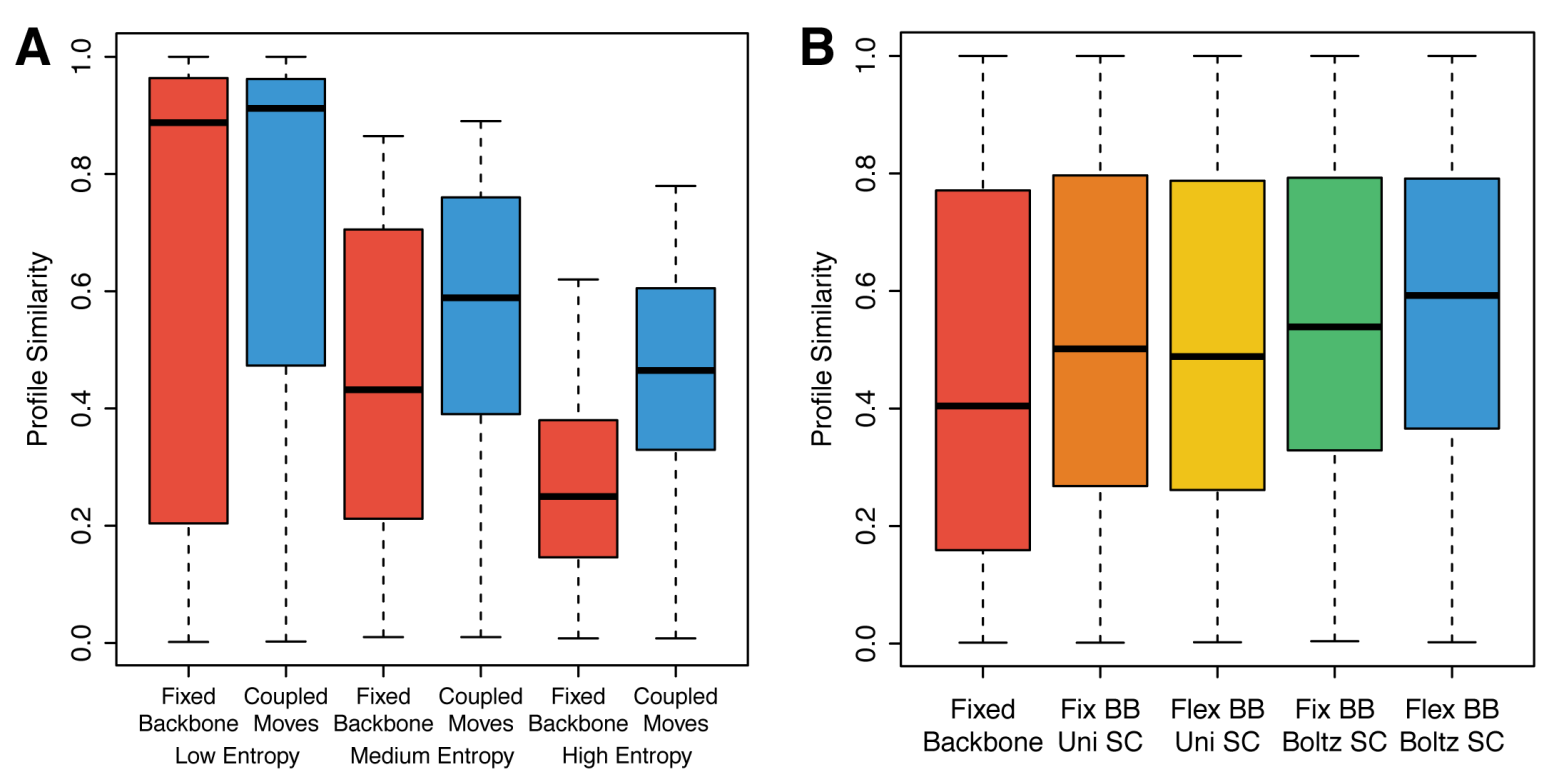
Sequence profile similarity distributions for different subsets of positions and for variations of the coupled moves method. A) Boxplots of profile similarity distributions for fixed backbone and coupled moves methods separated into three equal-sized groups based on sequence entropy in the natural sequences. B) Boxplots of profile similarity distributions for fixed backbone design and variations of the coupled moves method. Variants include using a Boltzmann distribution (“Boltz SC”) or a uniform distribution (“Uni SC”) to select mutations and side-chain conformations, and incorporating backbone flexibility (“Flex BB”) or using a fixed backbone (“Fix BB”).

To further understand the basis of this improvement, we divided the 158 positions into three groups based on the sequence entropy of each position in the natural families: high entropy (top third), medium entropy (middle third) and low entropy (bottom third). For each of these groups, we compared the profile similarity values for the coupled moves sequences and the fixed backbone sequences (**Fig 8A**). While the coupled moves sequences displayed higher median profile similarities for all groups relative to the fixed backbone sequences, the high entropy group yielded the greatest improvement, suggesting that the coupled moves method is better than fixed backbone design at accommodating multiple different amino acid residues at these positions. To determine whether or not the improvement in sequence profile prediction is simply due to increased sequence diversity, we calculated sequence profile similarity based on a null model that assumes a uniform amino acid distribution. While this also results in an improvement over fixed backbone design, it is still significantly lower in sequence profile similarity than sequences predicted using the coupled moves protocol (p < 10^−6^, **S10 Fig**).

To understand which components of the coupled moves method allowed it to achieve higher profile similarity to the natural ligand binding site sequences, we created several variants of the method based on how they select mutations and side-chain conformations and whether or not they allow backbone flexibility. Each variant was labeled “Boltz SC” or “Uni SC”, depending on whether it used a Boltzmann distribution or a uniform distribution to select mutations and side-chain conformations (see Methods), and “Flex BB” or “Fix BB”, depending on whether or not it allowed backbone flexibility. The profile similarity distribution for each variant is shown in **Fig 8B** compared to the standard coupled moves method (Flex BB, Boltz SC). Biasing the selection of mutations and side-chain conformations based on energy (Boltz SC) improved performance independently of whether or not backbone flexibility was allowed. However, backbone flexibility (Flex BB) only improved performance if a Boltzmann distribution was used for selecting mutations and side-chain conformations.

A possible explanation is that uniform selection in coupled flexible backbone design either leads to artificially collapsed structures (because smaller amino acids are more likely to be accepted in buried positions, which is then followed by backbone rearrangements around these smaller residues) or gives lower acceptance ratios. To examine these possibilities, we computed the percent glycine residues in sequences designed with each method variation as well as the acceptance ratio of all moves. We observed both a higher percentage of mutations to glycine and lower acceptance ratios using uniform selection of mutations and side-chain conformations (**S11 Fig**). These results highlight the advantage of biasing the selection of mutations and side-chain conformations based on energy distributions when performing flexible backbone protein design.

Finally, given the observation that up-weighting protein–ligand interactions improved the performance of the coupled moves method to predict specificity altering mutations, we predicted ligand binding site sequences by up-weighting protein–ligand interactions by a factor of 2 and 3. We found that up-weighting protein–ligand interactions resulted in lower profile similarity to naturally occurring binding site sequences (**S12 Fig**). These results may suggest that evolutionary selection pressures have constrained interactions between amino acid residues in these co-factor binding sites to a similar extent as interactions between amino acid residues and the small molecule co-factor.

## Discussion

In this study, we describe computational benchmarks to evaluate the accuracy of computational protein design for two important applications of protein engineering: 1) re-designing enzyme substrate specificity and 2) designing sequence libraries for protein–ligand interactions. We introduce a new computational protein design method that enables simultaneous sampling of protein backbone, amino acid side-chain and small molecule conformational flexibility, and we demonstrate that this method significantly improves both the accuracy of re-designing enzyme specificity and predicting sequence tolerance in ligand binding sites relative to fixed backbone design. These results show that subtle conformational changes in the protein backbone are important for accommodating mutations in ligand binding sites and that modeling these changes can improve the ability to design interactions between proteins and small molecules.

Despite the methodological advances described in this work, there exist a number of important limitations in the current methods that remain to be addressed. For example, it is highly unlikely that the presented approach can be used to predict the effect of mutations that are distant from the active site, given that allowed backbone flexibility is limited to small, local “backrub” moves. Allosteric mutations have recently been shown to be capable of altering the geometry between multiple subunits in protein–protein interactions [37] and may use a similar mechanism to modify interactions between proteins and small molecules. Modeling the effect of these mutations would require moving larger regions of the protein backbone, which could be accomplished by treating secondary structural elements as moveable rigid bodies connected by flexible linker regions. Such moves would need to be performed in a constrained manner such that they do not perturb important interactions in the active site that are required for catalysis. Moreover, our method does not model the chemical steps of an enzymatic reaction and how these steps might be affected by changes in the substrate. Processes involving bond breakage and formation could be addressed by quantum mechanical calculations.

Another limitation is the assumption that the protein remains fixed in length during the sequence design. Naturally occurring enzymes are not confined to a fixed sequence length and can acquire insertions and deletions in their active site loops to achieve altered specificities or even catalytic activities. This observation has previously been exploited to introduce new catalytic activities into an existing enzyme scaffold [38] and could potentially be a useful mechanism by which to design altered enzyme specificity. Active site loops whose lengths could be changed without disrupting protein stability could be identified prior to design, and moves that add or remove residues in these loops could be made using robotics-inspired loop modeling techniques such as kinematic closure [39].

Larger moves, such as insertions or deletions in active site loops or the re-arrangements between secondary structural elements described above, may be required to solve enzyme specificity re-design problems where the desired non-native substrate is significantly different in chemical structure from the native substrate. In the benchmark described in this study, we used example systems for which the native and non-native substrates shared a common substructure, allowing us to superimpose the non-native substrate onto the native substrate to create a starting model to use as input for design. If the substrates did not share a common substructure, more extensive remodeling of the active site may be necessary and ligand–protein docking may be required to obtain a model of the non-native substrate bound to the enzyme. Additionally, the implicit solvation model used in this study ignores the discrete size and asymmetry of water molecules and therefore cannot model water-mediated hydrogen bonding interactions. In subsequent work, the presented method could be used in combination with an explicit solvation model to more accurately capture water-mediated interactions between the ligand binding site residues and the small molecule.

The results of this study suggest that subtle changes to the protein backbone may be necessary for proteins to accommodate mutations that enable new functions, and that these mutations can successfully be accommodated via coupling “backrub” moves to changes in side-chain conformation. We find it notable that the same mechanisms of backbone movements commonly observed in protein structural heterogeneity [32] can be exploited to achieve altered functions. Our results thus support the idea that there are common mechanisms underlying protein dynamics and protein evolution [40], which has broad implications to the field of protein engineering and provides a promising route towards the development of computational models to predict how mutations affect protein function. We expect that future work on characterizing protein structural heterogeneity, for example by using room temperature X-ray crystallography [41], will provide valuable information on the types of motions that proteins undergo and enable us to take advantage of these motions when modeling and designing novel protein functions.

This study provides many examples where considerable changes in specificity can be made with one or two mutations while maintaining the catalytic activity of an enzyme. Altering specificity with a single mutation has recently been observed in interactions between PDZ domains and peptide ligands [13] and may provide an evolutionary mechanism by which proteins can obtain new functions without having to pass through an intermediate sequence with unfavorable fitness. Our study illustrates that these change of function mutations can be modeled and designed using computational protein design methods when subtle conformational changes of the protein backbone are allowed at the same time as sequence design. While this method has been specifically applied to interactions between proteins and small molecules in this study, the approach should be generally useful for any computational protein design problem. Finally, the benchmarks described in this study should enable further development and improvement in computational methods for re-designing enzyme specificity and designing sequence libraries for protein–ligand interactions.

## Methods

### Coupling backbone and side-chain flexibility

Backbone flexibility was modeled using three-residue “backrub” moves, which define a rotational axis between two Cα backbone atoms and rotate everything in between by an angle θ [25,32]. To determine a biophysically realistic distribution from which to sample θ, we created a dataset of 842 non-redundant high resolution (≤1.5Å) structures with a total of 2114 three-residue segments with alternate coordinates differing by greater than 0.2Å at Cα_i_ and less than 0.2Å at Cα_i-1_ and Cα_i+1_. We measured 2114 values of θ from this set of experimentally observed backrub motions and fit a Gaussian to this distribution, resulting in a standard deviation of 4.57° that we used as a default for the coupled moves method. Following each three-residue backrub move, we perform rotations of the two peptide bonds such that the displacement of the backbone C–O and N–H groups is minimized.

After the backbone move is completed, we iterate over each rotamer at position i and calculate its energy in the context of the new backbone conformation. Rotamers are generated using the Dunbrack backbone-dependent rotamer library [42]. A Boltzmann probability is calculated for each rotamer as follows:

$$P(E_{i})=\frac{1}{\sum_{i}e^{-\frac{E_{i}}{kT}}}e^{-\frac{E_{i}}{kT}}$$

where *E*_*i*_ is the difference in energy between rotamer i and the current rotamer. A rotamer is then selected using these probabilities. **S13 Fig** shows an example distribution of rotamer energies and their corresponding probabilities. If a position is a design position, one rotamer is selected for each amino acid, probabilities are computed for each amino acid based on the selected rotamers, and an amino acid is selected using these probabilities. A value of 0.6 was used for kT to calculate the probabilities. For the “Uni SC” protocol variant, rotamers and amino acids were selected using a uniform distribution. For the “Fix BB” protocol variant, the backbone moves were not performed.

### Coupling ligand rotation / translation and flexibility

Ligand rigid-body rotations and translations were sampled using two Gaussian distributions with a 1° standard deviation for rotations and a 0.1Å standard deviation for translations. After a rigid-body rotation and translation is completed, a rotamer is selected for the ligand using the same Boltzmann selection approach as for amino acid side chains described above. Ligand rotamers were generated using OpenEye OMEGA [43] with default parameters.

### Monte Carlo simulation

Coupled backbone / side-chain moves and coupled ligand rotation / translation and flexibility were combined in a Monte Carlo simulation using a constant temperature (kT = 0.6). Each move had a 90% probability of being a backbone / side-chain move and a 10% probability of being a ligand move. Each simulation was run for 1,000 moves and 20 simulations were run for each protein–ligand complex. All unique amino acid sequences accepted during each simulation were output into a FASTA file, and the resulting 20 FASTA files were filtered for redundancy and pooled into a single file for analysis. Command line arguments for running the coupled moves method in Rosetta are provided in **S1 Text**.

### Benchmark 1: Enzyme Specificity Altering Mutations

The command lines used to generate the results for benchmark 1 are shown in S1 Text. All positions with a side-chain heavy atom within 4.5Å of any atoms belonging to a substructure that differs between the native and non-native substrate were allowed to design to any amino acid. Neighboring positions were defined as any residue with a side-chain conformation that clashes (>5 Rosetta energy units) with a potential rotamer of a design position. All such neighboring positions were allowed to repack. Fixed backbone design was run to obtain the same number of total sequences as the coupled moves method.

The percent enrichment (PE) for each mutation was calculated as follows:

$$PE(WT\to MUT)={\%}_{non-native}–{\%}_{native}$$

$$PE(MUT\to WT)={\%}_{native}–{\%}_{non-native}$$

where %_*native*_ is the percent occurrence of the mutation in sequences designed for the native substrate/substrate analog and %_*non–native*_ is the percent occurrence of the mutation in sequences designed for the non-native substrate/substrate analog. *PE*(*WT* → *MUT*) was used for predictions that start with the wild-type structure and *PE*(*MUT* → *WT*) was used for predictions that start with the mutant structure.

A prediction was considered to be correct if it obtained a positive percent enrichment value. The “rank” of each mutation was determined by sorting all possible mutations at the given position in descending order of their percent enrichment values. We also used this sorted list to compute the percentile for each mutation. **S14 Fig** shows an example distribution of percent enrichment values for all mutations predicted for a given specificity switch.

### Benchmark 2: Ligand Binding Site Sequence Tolerance

The command lines used to generate the results for benchmark 2 are shown in S1 Text. All positions with a side-chain heavy atom within 6Å of any heavy atom on the ligand were allowed to design to any amino acid. Neighboring positions were defined as any residue with a side-chain conformation that clashes (>5 Rosetta energy units) with a potential rotamer of a design position. All such neighboring positions were allowed to repack. Fixed backbone design was run to obtain the same number of total sequences as the coupled moves method.

The profile similarity for each position was calculated as follows:

$$1-D^{JS}(p_{i},q_{i})$$

where p_i_ and q_i_ are the probability distributions over the 20 amino acids for the natural and designed sequences, respectively, at position i and D^JS^(x,y) is the Jensen–Shannon divergence between two distributions x and y, as described in [44].

The sequence entropy for each position was calculated as follows:

$$H_{i}=-\sum_{x}P_{x}\log_{20}P_{x}$$

where P_x_ is the percent of sequences with amino acid x at position i.

### Calculation of p-values

P-values for comparing the percent of correctly predicted specificity altering mutations in benchmark 1 were calculated using Fisher’s exact test. P-values for comparing the accuracy of predicting ligand binding site sequence profiles in benchmark 2 were calculated using a paired, two-tailed Student’s t-test assuming unequal variance.

## Supporting Information

S1 Fig Performance of predicting specificity altering mutations using the min packing method that minimizes torsions on side-chain rotamers during design. (TIF)

S2 Fig Performance of predicting specificity altering mutations using different weights for protein–ligand interactions. (TIF)

S3 Fig Comparison of fixed backbone and coupled moves methods based on RMSD from crystal structure of the residue mutated to change specificity as well as the surrounding neighboring residues. (TIF)

S4 Fig Sequence logos for the Short chain dehydrogenase binding site. (TIF)

S5 Fig Sequence logos for the Aminotransferase class I and II binding site. (TIF)

S6 Fig Sequence logos for the Methyltransferase domain binding site. (TIF)

S7 Fig Sequence logos for the Glutathione S-transferase binding site. (TIF)

S8 Fig Sequence logos for the Acetyltransferase (GNAT) binding site. (TIF)

S9 Fig Sequence logos for the Cytochrome P450 binding site. (TIF)

S10 Fig Comparison of the profile similarity distributions for fixed backbone design, the coupled moves method and a null model that assumes a uniform amino acid distribution. (TIF)

S11 Fig Comparison of the percent of glycine residues and acceptance ratios in sequences designed with fixed backbone design and variations of the coupled moves method. (TIF)

S12 Fig Performance of predicting ligand binding site sequences when up-weighting protein–ligand interactions. (TIF)

S13 Fig Example of the calculation of rotamer Boltzmann probabilities based on the distribution of rotamer energies. (TIF)

S14 Fig Example of the calculation of the percent enrichment in non-native sequences for predicted specificity altering mutations. Arrows indicate experimentally determined specificity altering mutations.
(TIF)

S1 Table Experimental data on enzyme substrate specificity altering mutations. For several of the enzymes in this table, the wild-type enzyme does not have detectable binding affinity for the non-native substrate. These cases are denoted by “Wild-type K_m_ nd”. Cases where the mutant enzyme did not have detectable binding affinity to the native substrate are denoted by “Mutant K_m_ nd.” Enzymes where binding affinities were not reported are labeled as “K_m_ nr”.
(DOCX)

S2 Table Comparison of fixed backbone and coupled moves methods on predicting specificity altering mutations starting from the wild-type enzyme (“WT to Mutant”). Dashes denote cases where the known mutation was not enriched in the predicted non-native substrate/substrate analog sequences and therefore not predicted to be a specificity altering mutation.
(DOCX)

S3 Table Comparison of fixed backbone and coupled moves methods on predicting specificity altering mutations starting from the mutant enzyme (“Mutant to WT”). Dashes denote cases where the known mutation was not enriched in the predicted native substrate/substrate analog sequences and therefore not predicted to be a specificity altering mutation.
(DOCX)

S1 Text Command line arguments for running the coupled moves method in Rosetta. (DOCX)
