# Supplementary figures and images for: Coupling Protein Side-Chain and Backbone Flexibility Improves the Re-design of Protein-Ligand Specificity
Source: PLoS Comput Biol. 2015 Sep 23;11(9):e1004335. doi: 10.1371/journal.pcbi.1004335 (PMC4580623; doi:10.1371/journal.pcbi.1004335)

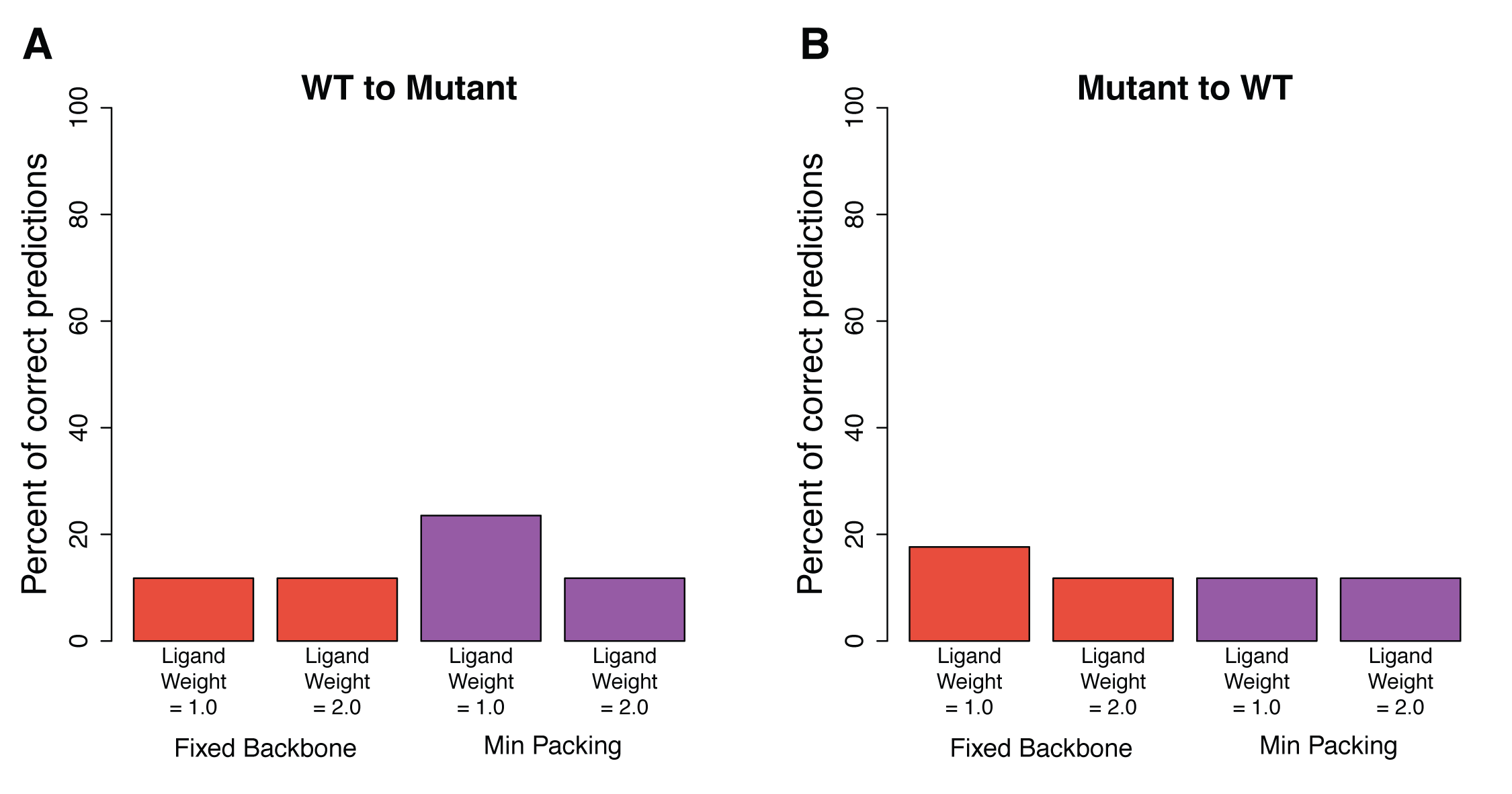

Supplement: S1 Fig — (TIF) [file pcbi.1004335.s001.tif]

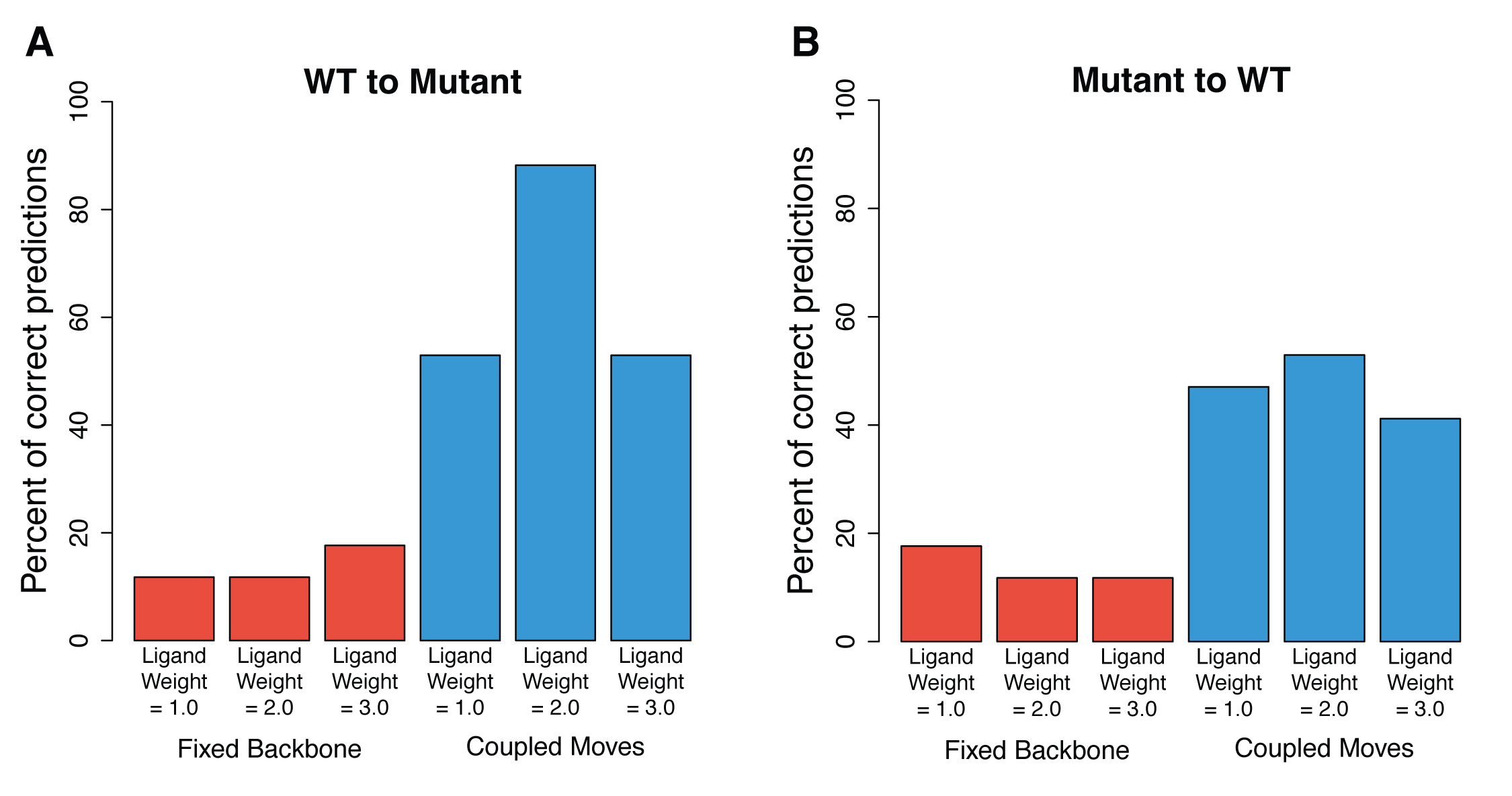

Supplement: S2 Fig — (TIF) [file pcbi.1004335.s002.tif]

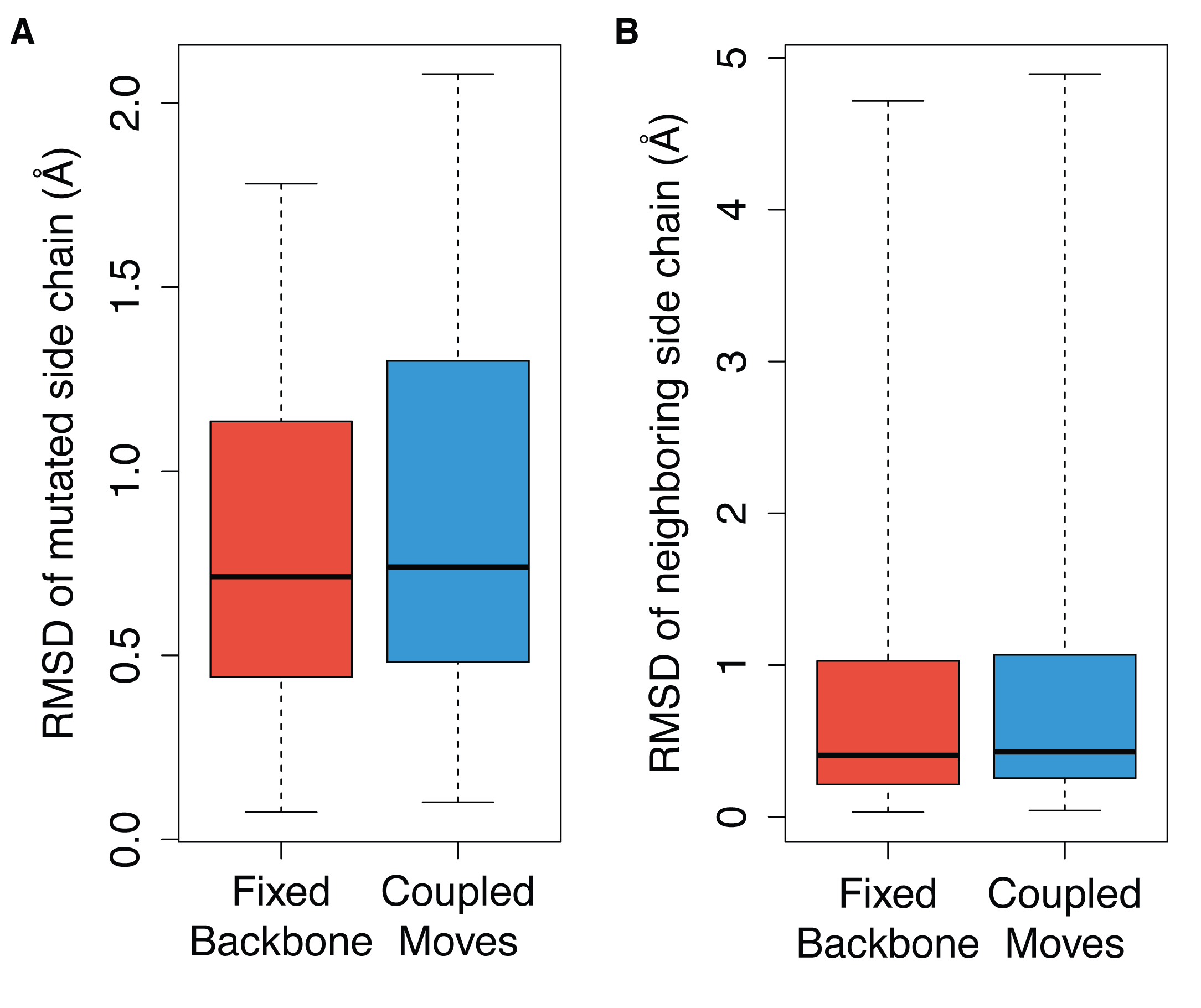

Supplement: S3 Fig — (TIF) [file pcbi.1004335.s003.tif]

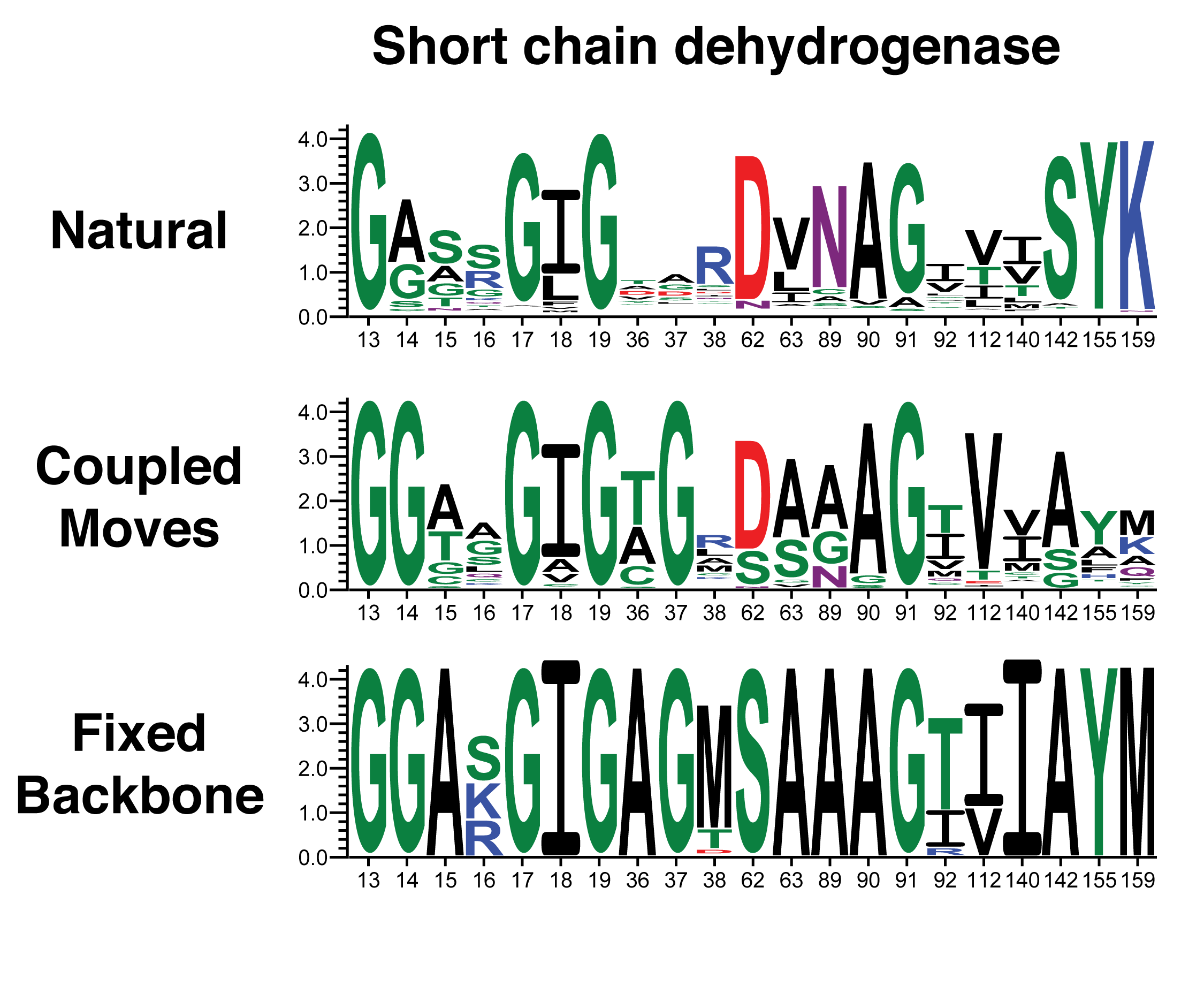

Supplement: S4 Fig — (TIF) [file pcbi.1004335.s004.tif]

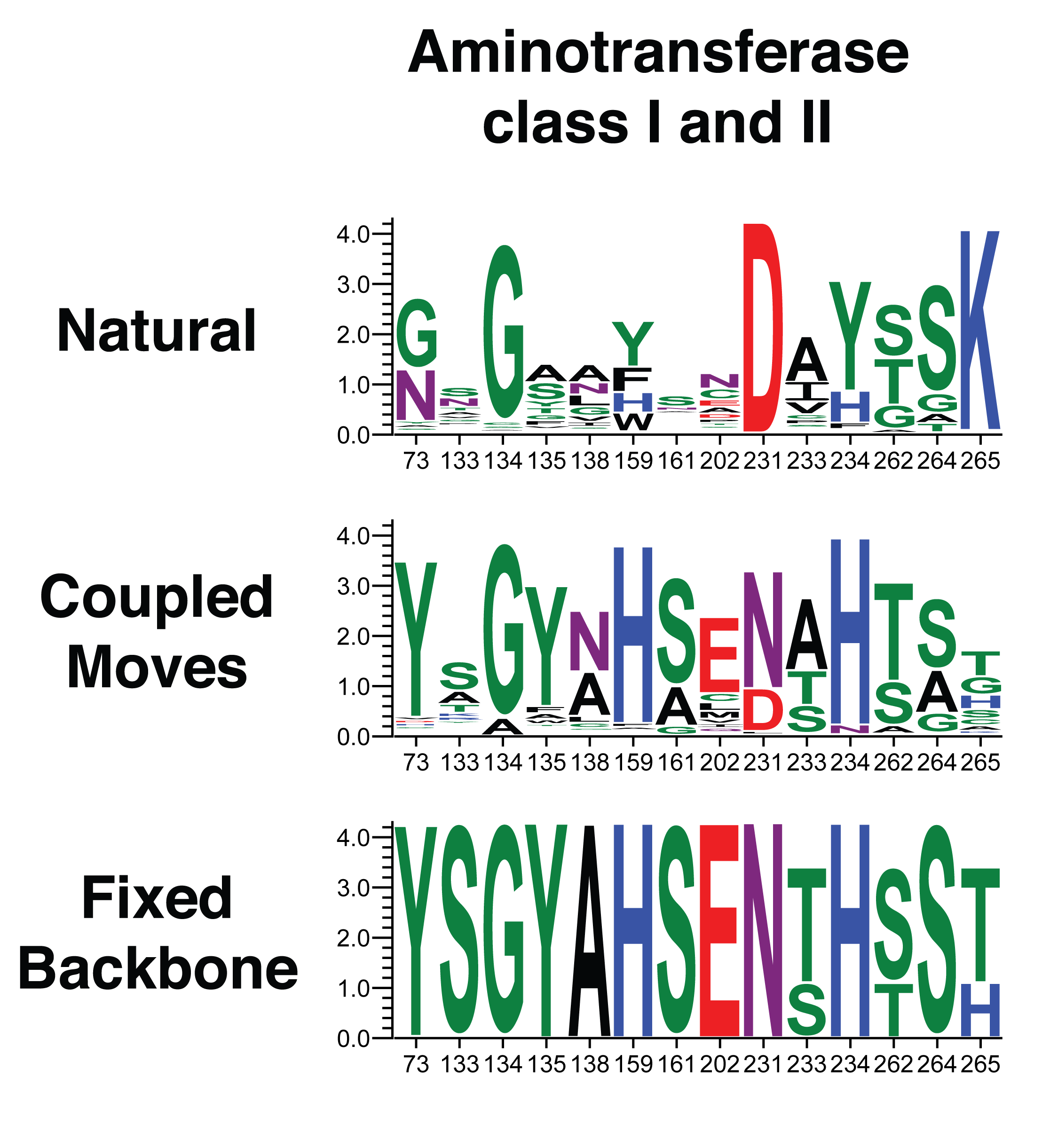

Supplement: S5 Fig — (TIF) [file pcbi.1004335.s005.tif]

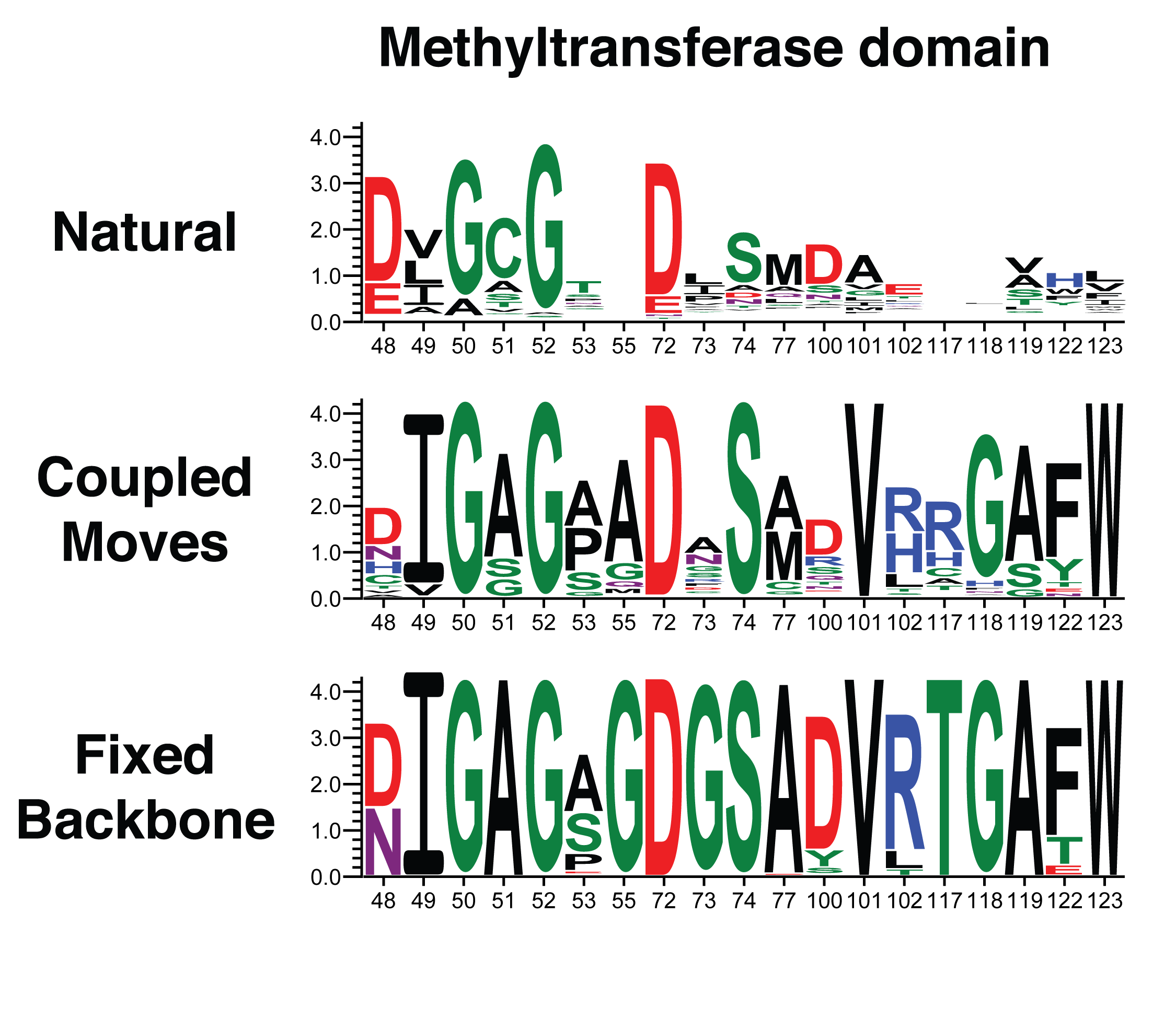

Supplement: S6 Fig — (TIF) [file pcbi.1004335.s006.tif]

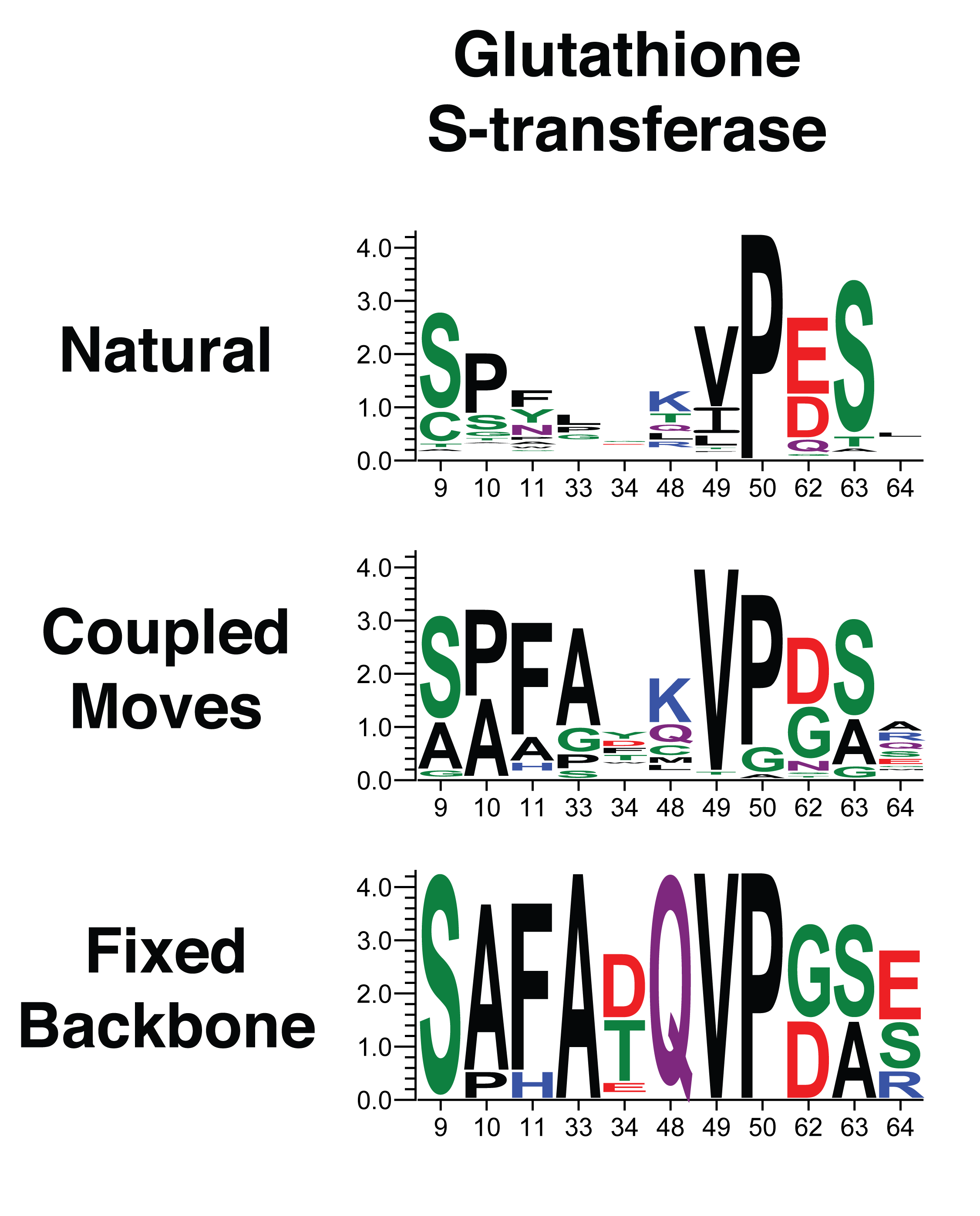

Supplement: S7 Fig — (TIF) [file pcbi.1004335.s007.tif]

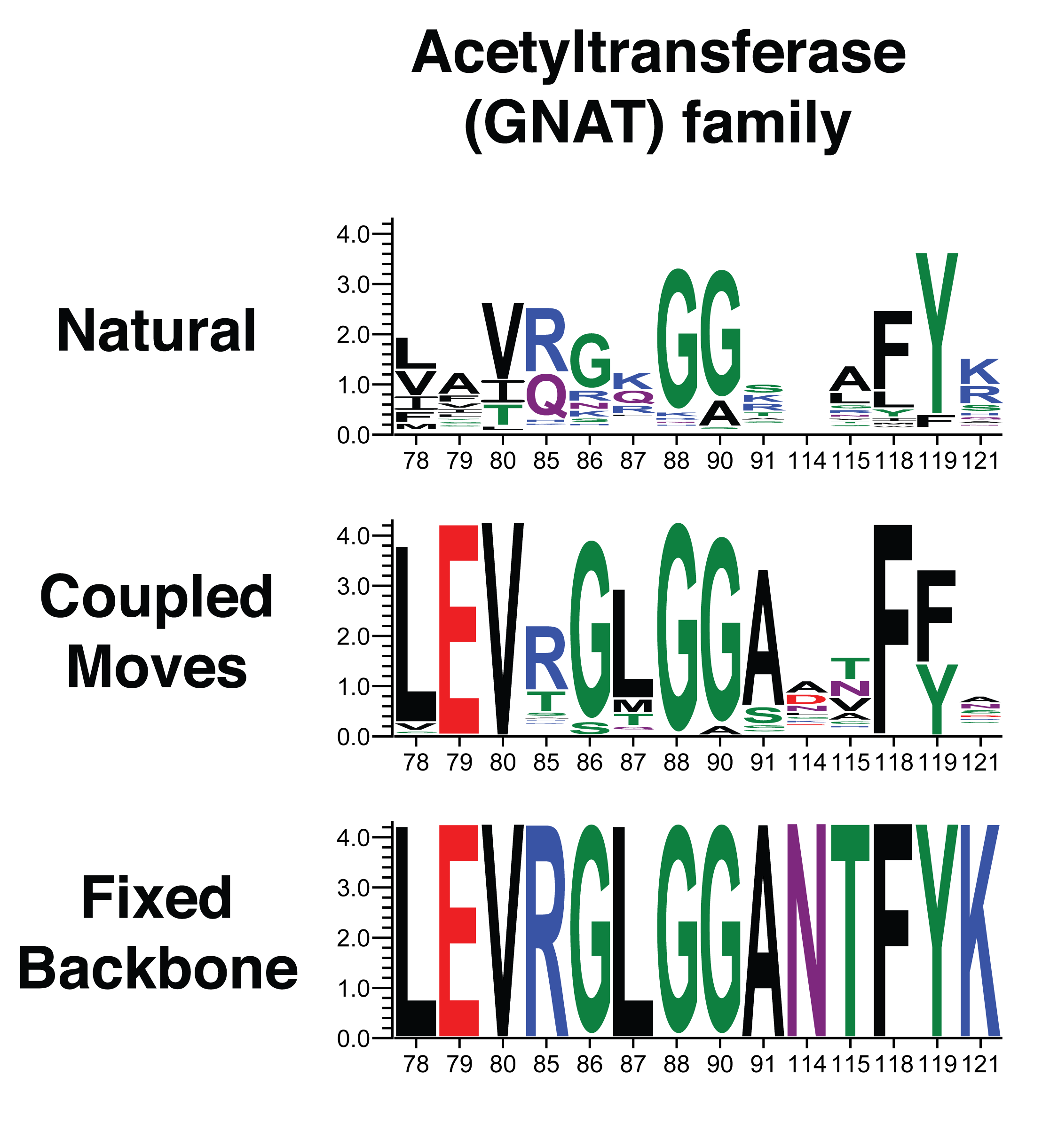

Supplement: S8 Fig — (TIF) [file pcbi.1004335.s008.tif]

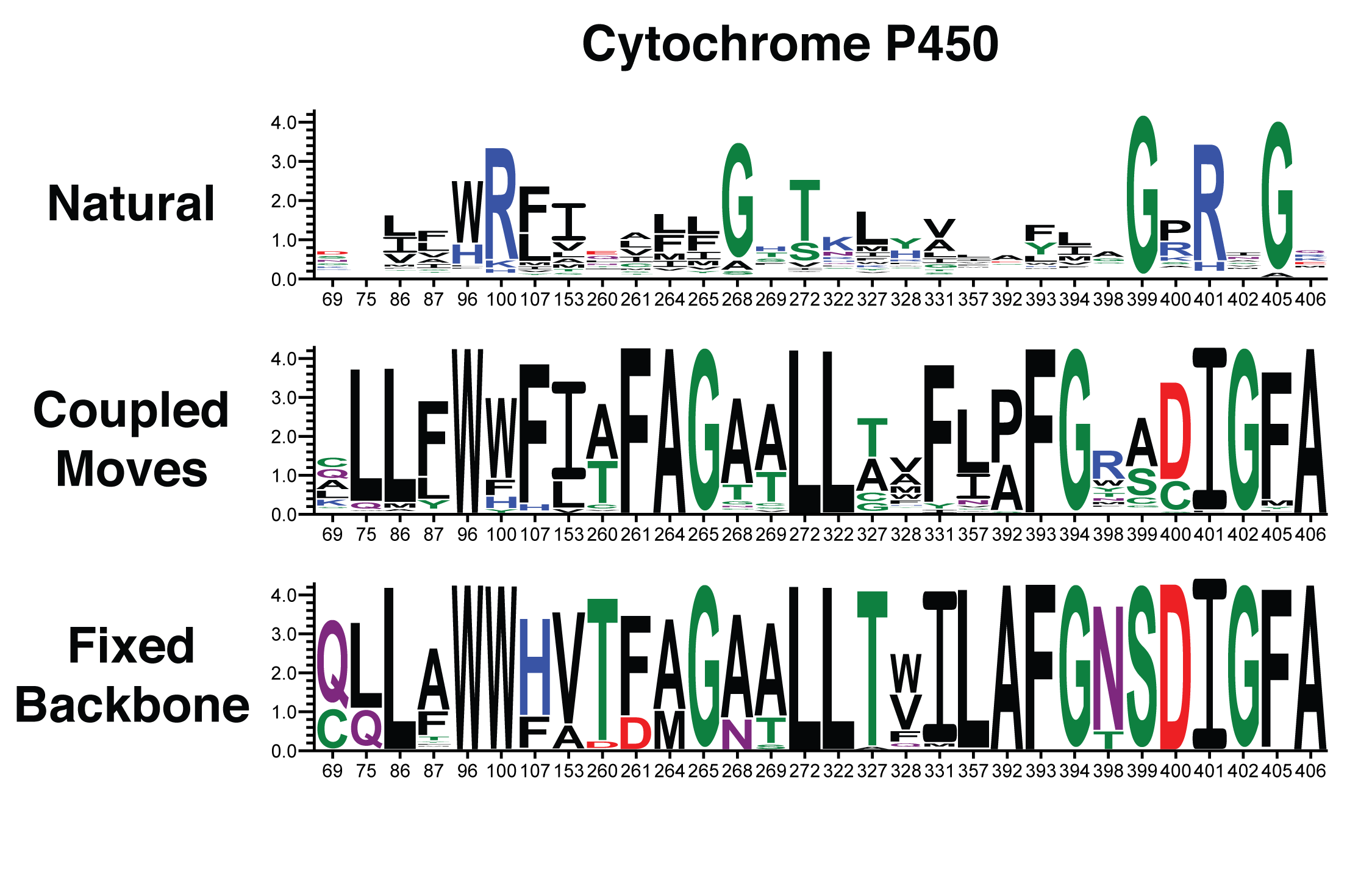

Supplement: S9 Fig — (TIF) [file pcbi.1004335.s009.tif]

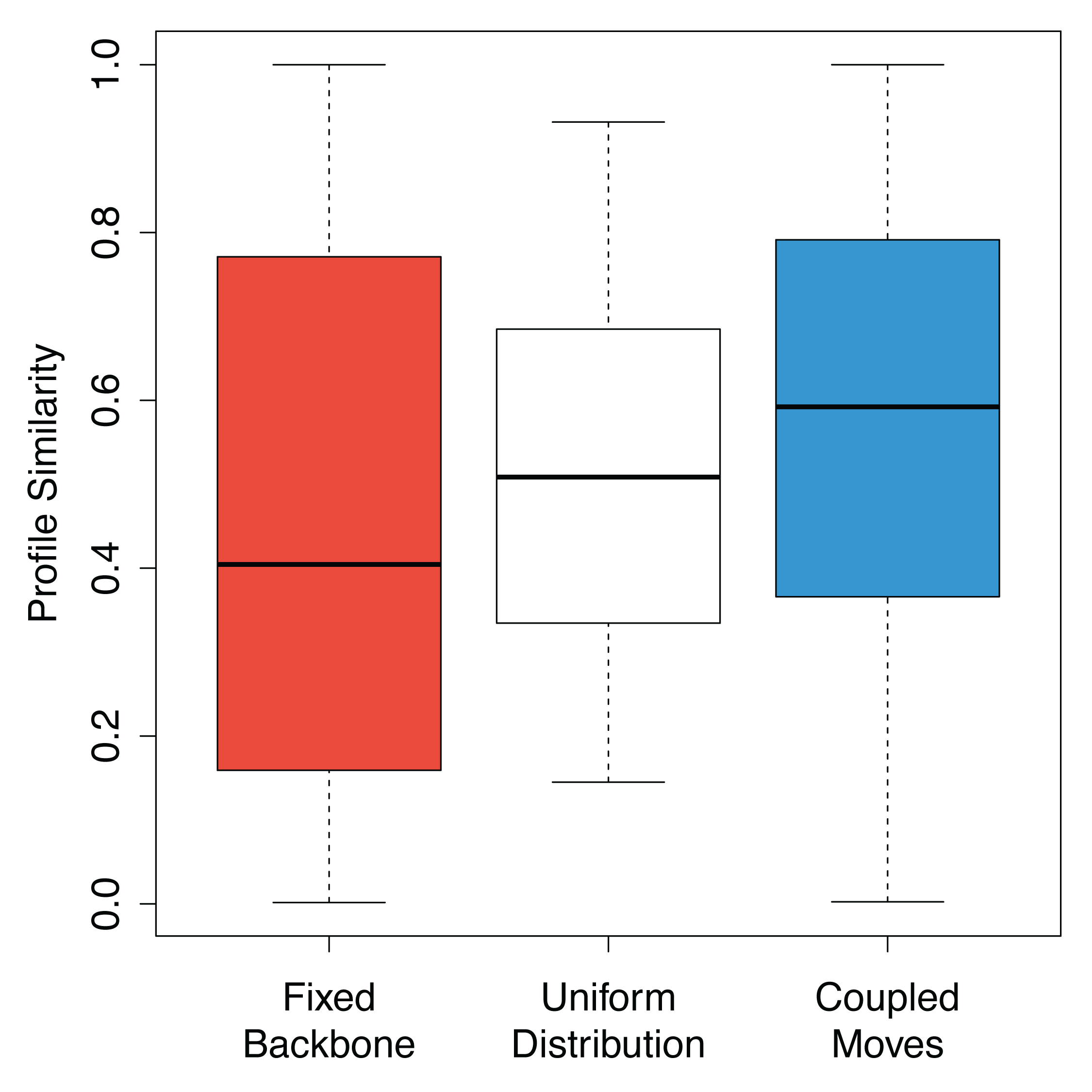

Supplement: S10 Fig — (TIF) [file pcbi.1004335.s010.tif]

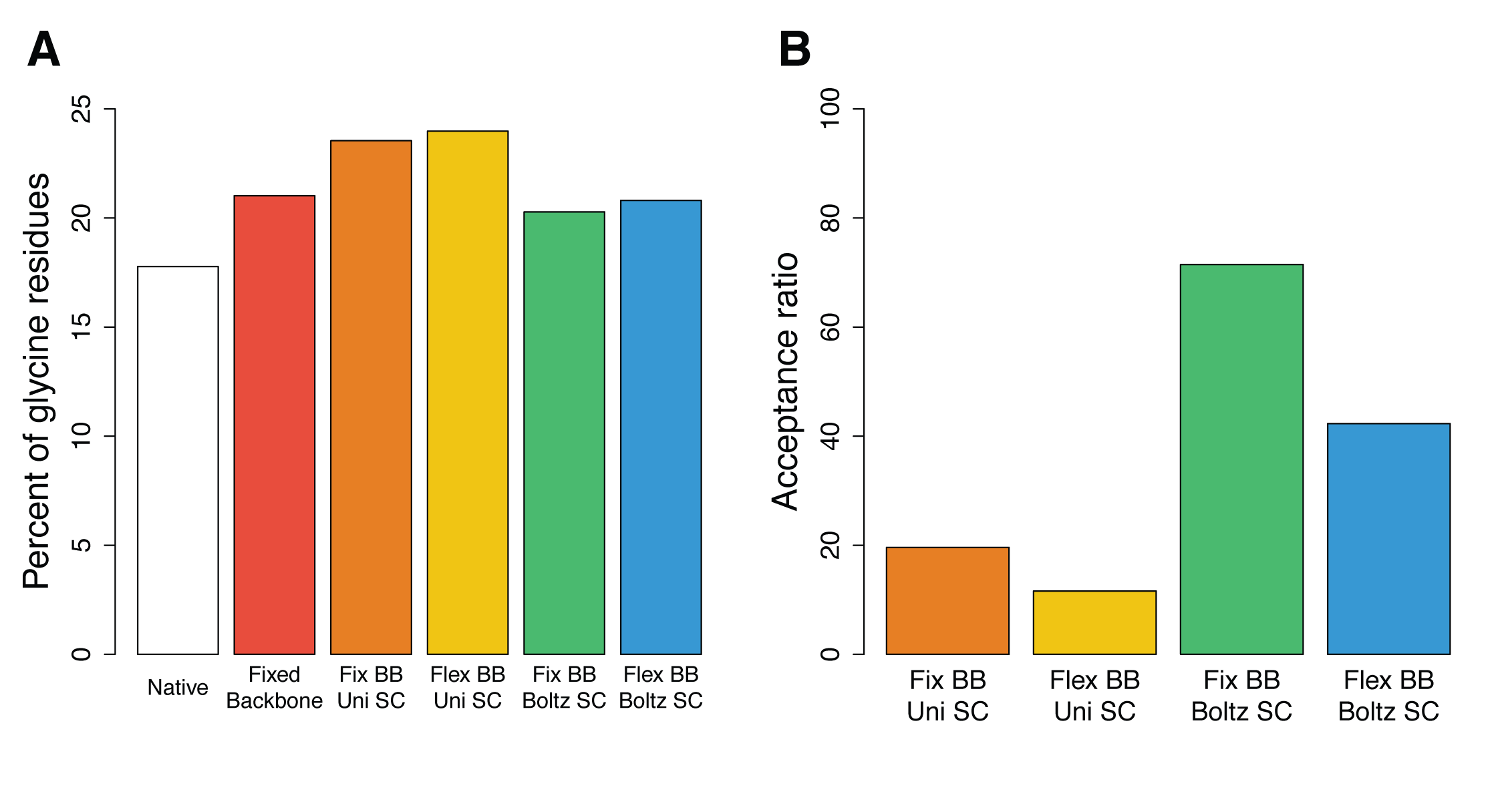

Supplement: S11 Fig — (TIF) [file pcbi.1004335.s011.tif]

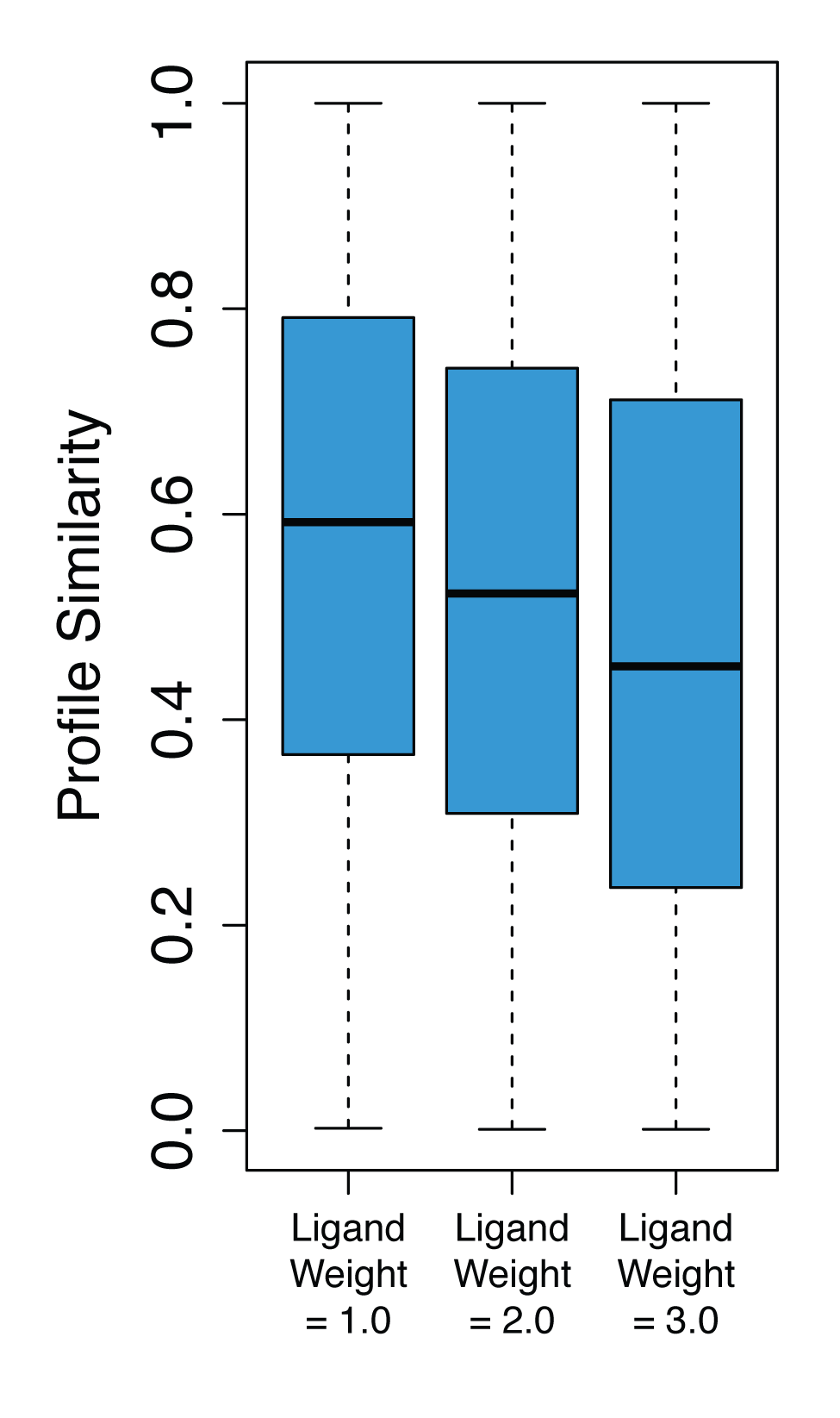

Supplement: S12 Fig — (TIF) [file pcbi.1004335.s012.tif]

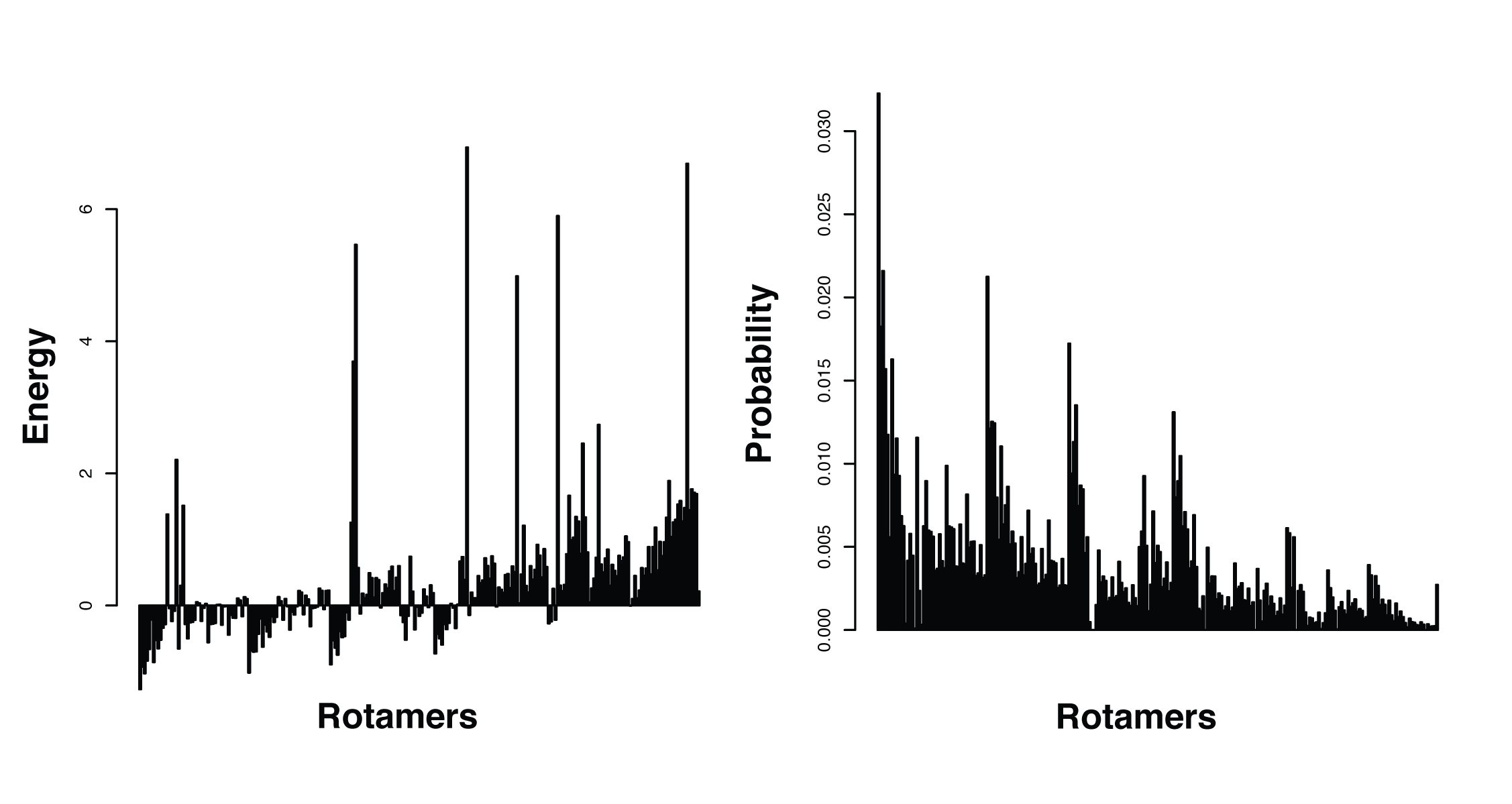

Supplement: S13 Fig — (TIF) [file pcbi.1004335.s013.tif]

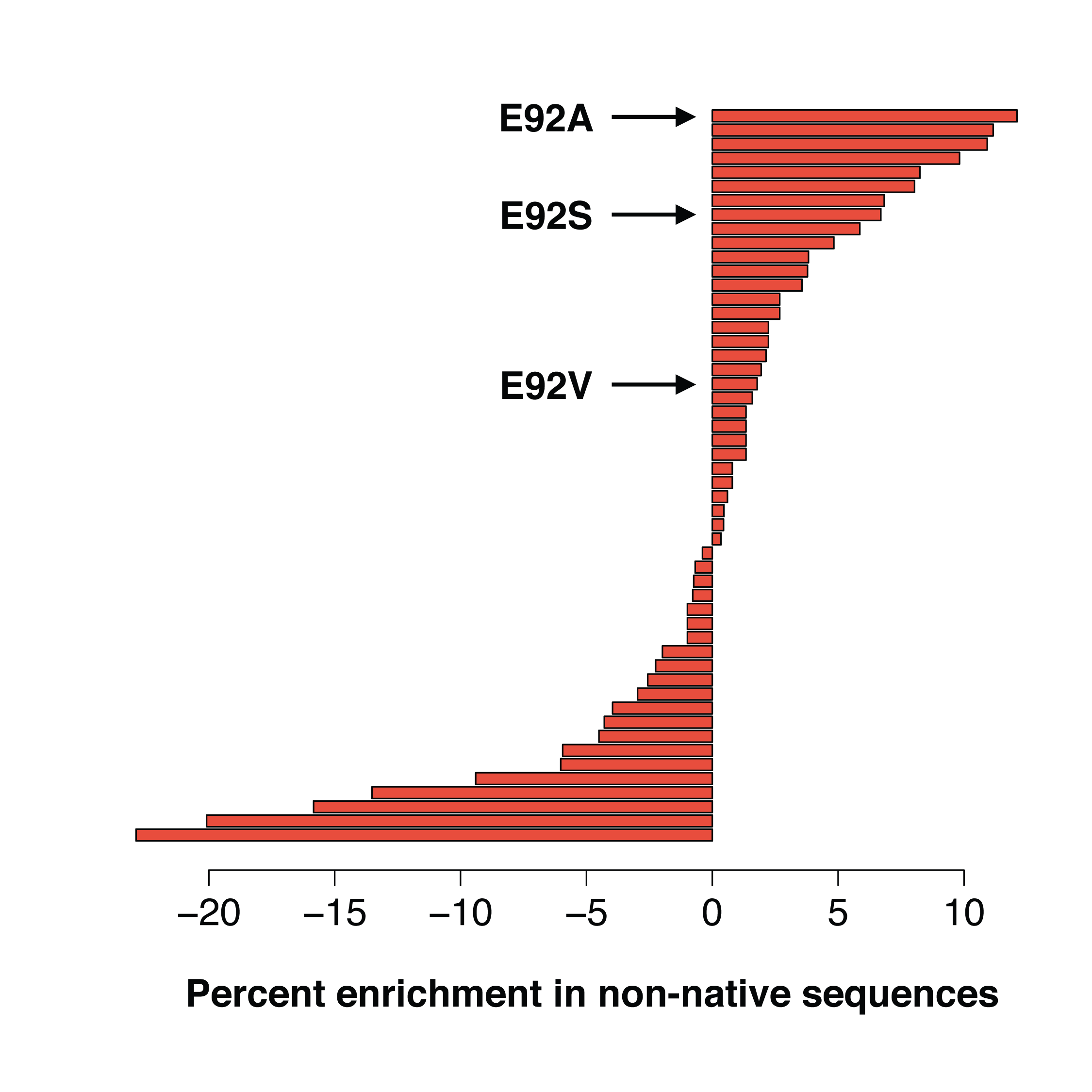

Supplement: S14 Fig — Arrows indicate experimentally determined specificity altering mutations. (TIF) [file pcbi.1004335.s014.tif]
