# Supplementary material for: Coupling Protein Side-Chain and Backbone Flexibility Improves the Re-design of Protein-Ligand Specificity
Source: PLoS Comput Biol. 2015 Sep 23;11(9):e1004335. doi: 10.1371/journal.pcbi.1004335 (PMC4580623; doi:10.1371/journal.pcbi.1004335)
Supplement: S1 Table — For several of the enzymes in this table, the wild-type enzyme does not have detectable binding affinity for the non-native substrate. These cases are denoted by “Wild-type Km nd”. Cases where the mutant enzyme did not have detectable binding affinity to the native substrate are denoted by “Mutant Km nd.” Enzymes where binding affinities were not reported are labeled as “Km nr”. (DOCX) [file pcbi.1004335.s015.docx]

**Table S1. Experimental data on enzyme substrate specificity altering mutations.**

| **Enzyme and citation** | **Native substrate** | **Non-native substrate** | **Mutation #** | **Wild-type PDB ID** | **Mutant PDB ID** | **Mutation** | **Fold change in native substrate K_m_ relative to wild-type** | **Fold change in non-native substrate K_m_ relative to wild-type** |
| --- | --- | --- | --- | --- | --- | --- | --- | --- |
| Proline dehydrogenase [1] | Proline | Hydroxy-proline | 1 | 2FZN | 3E2Q | Y540S | 0.59 | 3.95 |
| Flavocytochrome b2 [2] | L-lactate | L-mandelate | 2 | 1FCB | 1SZE | L230A | 0.08 | Wild-type K_m_ nd |
| N-acetyl-L-ornithine transcarbamylase [3] | N-acetyl-L-ornithine | N-succinyl-L-ornithine | 3 | 3KZO | 3L02 | E92A | K_m_ nr | K_m_ nr |
|  |  |  | 4 | 3KZO | 3L04 | E92S | K_m_ nr | K_m_ nr |
|  |  |  | 5 | 3KZO | 3L05 | E92P | K_m_ nr | K_m_ nr |
|  |  |  | 6 | 3KZO | 3L06 | E92V | K_m_ nr | K_m_ nr |
| Tyrosine ammonia-lyase [4] | L-Tyr | L-Phe | 7 | 2O7B | 2O78 | H89F | Mutant K_m_ nd | 26 |
| R-specific alcohol dehydrogenase [5] | NADP | NAD | 8 | 1ZK4 | 1ZK1 | G37D | Mutant K_m_ nd | Wild-type K_m_ nd |
| 2,5-Diketo-D-gluconic acid reductase [6] | NADPH | NADH | 9 | 1A80 | 1M9H | K232G | K_m_ nr | K_m_ nr |
|  |  |  | 10 | 1A80 | 1M9H | R238H | K_m_ nr | K_m_ nr |
| Purine nucleoside phosphorylase [7] | MeP-dR | Me(talo)-  MeP-R | 11 | 1PK7 | 1OUM | M64V | 0.042 | 1.36 |
| Cytosine deaminase [8] | Cytosine | 5-fluoro-cytosine | 12 | 1K70 | 1RA0 | D314S | 0.20 | 2.0 |
|  |  |  | 13 | 1K70 | 1RA5 | D314G | 0.67 | 3.3 |
|  |  |  | 14 | 1K70 | 1RAK | D314A | 0.09 | 0.9 |
| Farnesyltransferase [9] | Farnesyl | Geranyl-geranyl | 15 | 2H6F | 2H6G | W602T | Mutant K_m_ nd | Wild-type K_m_ nd |
| Alpha-galactosidase [10] | Galactose | N-acetyl-galactosamine | 16 | 3HG5 | 3LX9 | E203S | 0.14 | Wild-type K_m_ nd |
|  |  |  | 17 | 3HG5 | 3LX9 | L206A |  |  |

For several of the enzymes in this table, the wild-type enzyme does not have detectable binding affinity for the non-native substrate. These cases are denoted by “Wild-type K_m_ nd”. Cases where the mutant enzyme did not have detectable binding affinity to the native substrate are denoted by “Mutant K_m_ nd.” Enzymes where binding affinities were not reported are labeled as “K_m_ nr”.

**References**

1. Ostrander EL, Larson JD, Schuermann JP, Tanner JJ (2009) A conserved active site tyrosine residue of proline dehydrogenase helps enforce the preference for proline over hydroxyproline as the substrate. Biochemistry 48: 951–959. doi:10.1021/bi802094k.

2. Mowat CG, Wehenkel A, Green AJ, Walkinshaw MD, Reid GA, et al. (2004) Altered substrate specificity in flavocytochrome b2: structural insights into the mechanism of L-lactate dehydrogenation. Biochemistry 43: 9519–9526. doi:10.1021/bi049263m.

3. Shi D, Yu X, Cabrera-Luque J, Chen TY, Roth L, et al. (2007) A single mutation in the active site swaps the substrate specificity of N-acetyl-L-ornithine transcarbamylase and N-succinyl-L-ornithine transcarbamylase. Protein Sci 16: 1689–1699. doi:10.1110/ps.072919907.

4. Louie GV, Bowman ME, Moffitt MC, Baiga TJ, Moore BS, et al. (2006) Structural determinants and modulation of substrate specificity in phenylalanine-tyrosine ammonia-lyases. Chemistry & Biology 13: 1327–1338. doi:10.1016/j.chembiol.2006.11.011.

5. Schlieben NH, Niefind K, Müller J, Riebel B, Hummel W, et al. (2005) Atomic resolution structures of R-specific alcohol dehydrogenase from Lactobacillus brevis provide the structural bases of its substrate and cosubstrate specificity. Journal of Molecular Biology 349: 801–813. doi:10.1016/j.jmb.2005.04.029.

6. Sanli G, Banta S, Anderson S, Blaber M (2004) Structural alteration of cofactor specificity in Corynebacterium 2,5‐diketo‐D‐gluconic acid reductase. Protein Science 13: 504–512. doi:10.1110/ps.03450704.

7. Bennett EM, Anand R, Allan PW, Hassan AEA, Hong JS, et al. (2003) Designer Gene Therapy Using an Escherichia coli Purine Nucleoside Phosphorylase/Prodrug System. Chemistry & Biology 10: 1173–1181. doi:10.1016/j.chembiol.2003.11.008.

8. Mahan SD, Ireton GC, Knoeber C, Stoddard BL, Black ME (2004) Random mutagenesis and selection of Escherichia coli cytosine deaminase for cancer gene therapy. Protein Engineering Design and Selection 17: 625–633. doi:10.1093/protein/gzh074.

9. Terry KL, Casey PJ, Beese LS (2006) Conversion of protein farnesyltransferase to a geranylgeranyltransferase. Biochemistry 45: 9746–9755. doi:10.1021/bi060295e.

10. Tomasic IB, Metcalf MC, Guce AI, Clark NE, Garman SC (2010) Interconversion of the specificities of human lysosomal enzymes associated with Fabry and Schindler diseases. J Biol Chem 285: 21560–21566. doi:10.1074/jbc.M110.118588.
