# Supplementary material for: Coupling Protein Side-Chain and Backbone Flexibility Improves the Re-design of Protein-Ligand Specificity
Source: PLoS Comput Biol. 2015 Sep 23;11(9):e1004335. doi: 10.1371/journal.pcbi.1004335 (PMC4580623; doi:10.1371/journal.pcbi.1004335)
Supplement: S2 Table — Dashes denote cases where the known mutation was not enriched in the predicted non-native substrate/substrate analog sequences and therefore not predicted to be a specificity altering mutation. (DOCX) [file pcbi.1004335.s016.docx]

**Table S2. Comparison of fixed backbone and coupled moves methods on predicting specificity altering mutations starting from the wild-type enzyme (“WT to Mutant”).**

|  | **Fixed Backbone Design** | | | | **Coupled Moves Method** | | | |
| --- | --- | --- | --- | --- | --- | --- | --- | --- |
|  | **Ligand Weight = 1.0** | | **Ligand Weight = 2.0** | | **Ligand Weight = 1.0** | | **Ligand Weight = 2.0** | |
| **Mutant #** | **Percentile** | **Rank** | **Percentile** | **Rank** | **Percentile** | **Rank** | **Percentile** | **Rank** |
| 1 | – | – | – | – | 96.0 | 2 | 95.8 | 2 |
| 2 | – | – | – | – | 80.8 | 15 | 63.0 | 28 |
| 3 | 81.8 | 5 | 78.9 | 5 | 97.8 | 2 | 100 | 1 |
| 4 | – | – | – | – | 80.4 | 10 | 86.5 | 8 |
| 5 | – | – | – | – | – | – | – | – |
| 6 | – | – | – | – | – | – | 63.5 | 20 |
| 7 | – | – | – | – | 62.5 | 10 | 90.9 | 3 |
| 8 | 38.9 | 12 | 93.8 | 2 | 80.0 | 12 | 90.2 | 6 |
| 9 | – | – | – | – | – | – | 71.4 | 19 |
| 10 | – | – | – | – | – | – | – | – |
| 11 | – | – | – | – | 100 | 1 | 69.2 | 13 |
| 12 | – | – | – | – | – | – | 63.6 | 9 |
| 13 | – | – | – | – | – | – | 90.9 | 3 |
| 14 | – | – | – | – | 96.4 | 2 | 72.7 | 7 |
| 15 | – | – | – | – | – | – | 61.3 | 37 |
| 16 | – | – | – | – | – | – | 93.5 | 7 |
| 17 | – | – | – | – | 100 | 1 | 87.1 | 13 |

Dashes denote cases where the known mutation was not enriched in the predicted non-native substrate/substrate analog sequences and therefore not predicted to be a specificity altering mutation.
