# Supplementary material for: Coupling Protein Side-Chain and Backbone Flexibility Improves the Re-design of Protein-Ligand Specificity
Source: PLoS Comput Biol. 2015 Sep 23;11(9):e1004335. doi: 10.1371/journal.pcbi.1004335 (PMC4580623; doi:10.1371/journal.pcbi.1004335)
Supplement: S3 Table — Dashes denote cases where the known mutation was not enriched in the predicted native substrate/substrate analog sequences and therefore not predicted to be a specificity altering mutation. (DOCX) [file pcbi.1004335.s017.docx]

**Table S3. Comparison of fixed backbone and coupled moves methods on predicting specificity altering mutations starting from the mutant enzyme (“Mutant to WT”).**

|  | **Fixed Backbone Design** | | | | **Coupled Moves Method** | | | |
| --- | --- | --- | --- | --- | --- | --- | --- | --- |
|  | **Ligand Weight = 1.0** | | **Ligand Weight = 2.0** | | **Ligand Weight = 1.0** | | **Ligand Weight = 2.0** | |
| **Mutant #** | **Percentile** | **Rank** | **Percentile** | **Rank** | **Percentile** | **Rank** | **Percentile** | **Rank** |
| 1 | – | – | – | – | – | – | – | – |
| 2 | – | – | – | – | 100 | 1 | 97.8 | 3 |
| 3 | – | – | – | – | – | – | – | – |
| 4 | – | – | – | – | 92.7 | 5 | 60.4 | 20 |
| 5 | – | – | – | – | 64.3 | 16 | 34.3 | 24 |
| 6 | 66.7 | 5 | – | – | 94.6 | 5 | 85.5 | 11 |
| 7 | – | – | – | – | – | – | 78.6 | 7 |
| 8 | 100 | 1 | 96.0 | 2 | 100 | 1 | 100 | 1 |
| 9 | – | – | – | – | – | – | – | – |
| 10 | – | – | – | – | – | – | – | – |
| 11 | – | – | – | – | 77.8 | 9 | 73.5 | 10 |
| 12 | – | – | – | – | – | – | 60.7 | 12 |
| 13 | – | – | – | – | – | – | – | – |
| 14 | – | – | – | – | – | – | – | – |
| 15 | – | – | – | – | – | – | – | – |
| 16 | – | – | – | – | 76.5 | 17 | – | – |
| 17 | – | – | – | – | – | – | – | – |

Dashes denote cases where the known mutation was not enriched in the predicted native substrate/substrate analog sequences and therefore not predicted to be a specificity altering mutation.
