# Supplementary material for: Coupling Protein Side-Chain and Backbone Flexibility Improves the Re-design of Protein-Ligand Specificity
Source: PLoS Comput Biol. 2015 Sep 23;11(9):e1004335. doi: 10.1371/journal.pcbi.1004335 (PMC4580623; doi:10.1371/journal.pcbi.1004335)
Supplement: S1 Text — (DOCX) [file pcbi.1004335.s018.docx]

**Benchmark 1: Predicting Enzyme Specificity Altering Mutations**

Command line arguments for coupled moves method:

~/Rosetta/main/source/bin/coupled_moves.linuxgccrelease

–s pdb_file –resfile res_file –database ~/Rosetta/main/database –mute protocols.backrub.BackrubMover –extra_res_fa params_file –ex1 –ex2

–extrachi_cutoff 0 –nstruct 20 –coupled_moves::mc_kt 0.6 –coupled_moves::ntrials 1000 –coupled_moves::initial_repack false –coupled_moves::ligand_mode true

–coupled_moves::ligand_weight N

where pdb_file is the input starting structure, res_file is the file specifying the repack and design positions, params_file is the ligand parameter file and N is the weight on protein – ligand interactions. This produced 20 FASTA files that were filtered for redundancy and pooled into a single file to use for analysis.

Command line arguments for fixed backbone design:

~/Rosetta/main/source/bin/coupled_moves.linuxgccrelease –s pdb_file –resfile res_file –database ~/Rosetta/main/database –mute protocols.backrub.BackrubMover –extra_res_fa params_file –ex1 –ex2

–extrachi_cutoff 0 –nstruct 1 –coupled_moves::mc_kt 0.6 –coupled_moves::ntrials 0 –coupled_moves::initial_repack true –coupled_moves::ligand_mode true

–coupled_moves::ligand_weight N

where pdb_file, res_file, params_file and N are as described above. This command line forces the coupled moves method to perform fixed backbone repacking and designing only, without performing any coupled moves, and results in one sequence per run. This was run repeatedly until the number of total sequences was equal to the number of sequences output by the coupled moves method.

**Benchmark 2: Ligand Binding Site Sequence Tolerance**

Command line arguments for standard coupled moves method using flexible backbone moves and Boltzmann weighted rotamer selection (“Flex Boltz”):

~/Rosetta/main/source/bin/coupled_moves.linuxgccrelease –s pdb_file –resfile res_file –database ~/Rosetta/main/database –mute protocols.backrub.BackrubMover –extra_res_fa params_file –ex1 –ex2 –extrachi_cutoff 0 –nstruct 20 –coupled_moves::mc_kt 0.6 –coupled_moves::ntrials 1000 –coupled_moves::initial_repack true –coupled_moves::ligand_mode true –coupled_moves::fix_backbone false –coupled_moves::bias_sampling true –coupled_moves::bump_check true –coupled_moves::ligand_weight 1.0

Command line arguments for coupled moves method using flexible backbone moves and uniform rotamer selection (“Flex Uni”):

~/Rosetta/main/source/bin/coupled_moves.linuxgccrelease –s pdb_file –resfile res_file –database ~/Rosetta/main/database –mute protocols.backrub.BackrubMover –extra_res_fa params_file –ex1 –ex2 –extrachi_cutoff 0 –nstruct 20 –coupled_moves::mc_kt 0.6 –coupled_moves::ntrials 1000 –coupled_moves::initial_repack true –coupled_moves::ligand_mode true –coupled_moves::fix_backbone false –coupled_moves::bias_sampling false –coupled_moves::bump_check false –coupled_moves::ligand_weight 1.0

Command line arguments for coupled moves method with fixed backbone and Boltzmann weighted rotamer selection (“Fix Boltz”):

~/Rosetta/main/source/bin/coupled_moves.linuxgccrelease –s pdb_file –resfile res_file –database ~/Rosetta/main/database –mute protocols.backrub.BackrubMover –extra_res_fa params_file –ex1 –ex2 –extrachi_cutoff 0 –nstruct 20 –coupled_moves::mc_kt 0.6 –coupled_moves::ntrials 1000 –coupled_moves::initial_repack true –coupled_moves::ligand_mode true –coupled_moves::fix_backbone true –coupled_moves::bias_sampling true –coupled_moves::bump_check true –coupled_moves::ligand_weight 1.0

Command line arguments for coupled moves method with fixed backbone and uniform rotamer selection (“Fix Uni”):

~/Rosetta/main/source/bin/coupled_moves.linuxgccrelease –s pdb_file –resfile res_file –database ~/Rosetta/main/database –mute protocols.backrub.BackrubMover –extra_res_fa params_file –ex1 –ex2 –extrachi_cutoff 0 –nstruct 20 –coupled_moves::mc_kt 0.6 –coupled_moves::ntrials 1000 –coupled_moves::initial_repack true –coupled_moves::ligand_mode true –coupled_moves::fix_backbone true –coupled_moves::bias_sampling false –coupled_moves::bump_check false –coupled_moves::ligand_weight 1.0

Command line arguments for fixed backbone design:

~/Rosetta/main/source/bin/coupled_moves.linuxgccrelease –s pdb_file –resfile res_file –database ~/Rosetta/main/database –mute protocols.backrub.BackrubMover –extra_res_fa params_file –ex1 –ex2

–extrachi_cutoff 0 –nstruct 1 –coupled_moves::mc_kt 0.6 –coupled_moves::ntrials 0 –coupled_moves::initial_repack true –coupled_moves::ligand_mode true

–coupled_moves::ligand_weight 1.0

As with benchmark 1, fixed backbone design was run repeatedly until the number of total sequences was equal to the number of sequences output by the standard coupled moves method (“Flex Boltz”).

Rosetta version 57341 was used to obtain the data presented in the manuscript.
